# Individualized Interactomes From Pulmonary Arterial Hypertension Cell Biopsies Inform Pharmacotherapeutic Response

**DOI:** 10.1016/j.jacbts.2026.101655

**Published:** 2026-08-18

**Authors:** Rui-Sheng Wang, Navneet Singh, Henri Wathieu, Grayson Baird, Katherine Cox-Flaherty, James R. Klinger, Christopher J. Mullin, Mandy Pereira, Mary Whittenhall, Elizabeth O. Harrington, Bradley A. Maron, Corey E. Ventetuolo

**Affiliations:** a Department of Medicine, Brigham and Women’s Hospital, Boston, Massachusetts, USA; b Department of Medicine, Alpert Medical School of Brown University, Providence, Rhode Island, USA; c Department of Diagnostic Imaging, Alpert Medical School of Brown University and Brown University Health, Providence, Rhode Island, USA; d Inova Fairfax, Falls Church, Virginia, USA; e Department of Medicine, University of Maryland School of Medicine, Baltimore, Maryland, USA; f Institute for Health Computing, University of Maryland, Bethesda, Maryland, USA; g Department of Health Services, Policy and Practice, Brown University, Providence, Rhode Island, USA

**Keywords:** biopsy, endothelial dysfunction, network medicine, precision medicine, pulmonary arterial hypertension

## Abstract

Pulmonary arterial hypertension (PAH) is characterized by molecular heterogeneity, which has limited personalized approaches to treatment selection. In this study, the authors assembled a novel pipeline that leveraged pulmonary artery endothelial cell biopsies acquired at the point of care to build individualized interactomes that served as the basis for a systems pharmacology analysis. Concordance between the molecular targets of a prescribed PAH pharmacotherapy class and interactome topology was associated with improved clinical outcomes using a retrospective in silico trial design. These data suggest that clinically actionable individualized treatment selection is feasible in PAH with relevance to other complex cardiovascular diseases.

## Highlights

• Network analysis of PAEC biopsy transcriptomics individualized PAH pathobiology.
• Systems pharmacology analysis of patient-specific interactomes informed PAH outcomes.
• This study provides a clinically actionable pipeline to individualize PAH treatment.

## Visual Abstract

**Figure:**
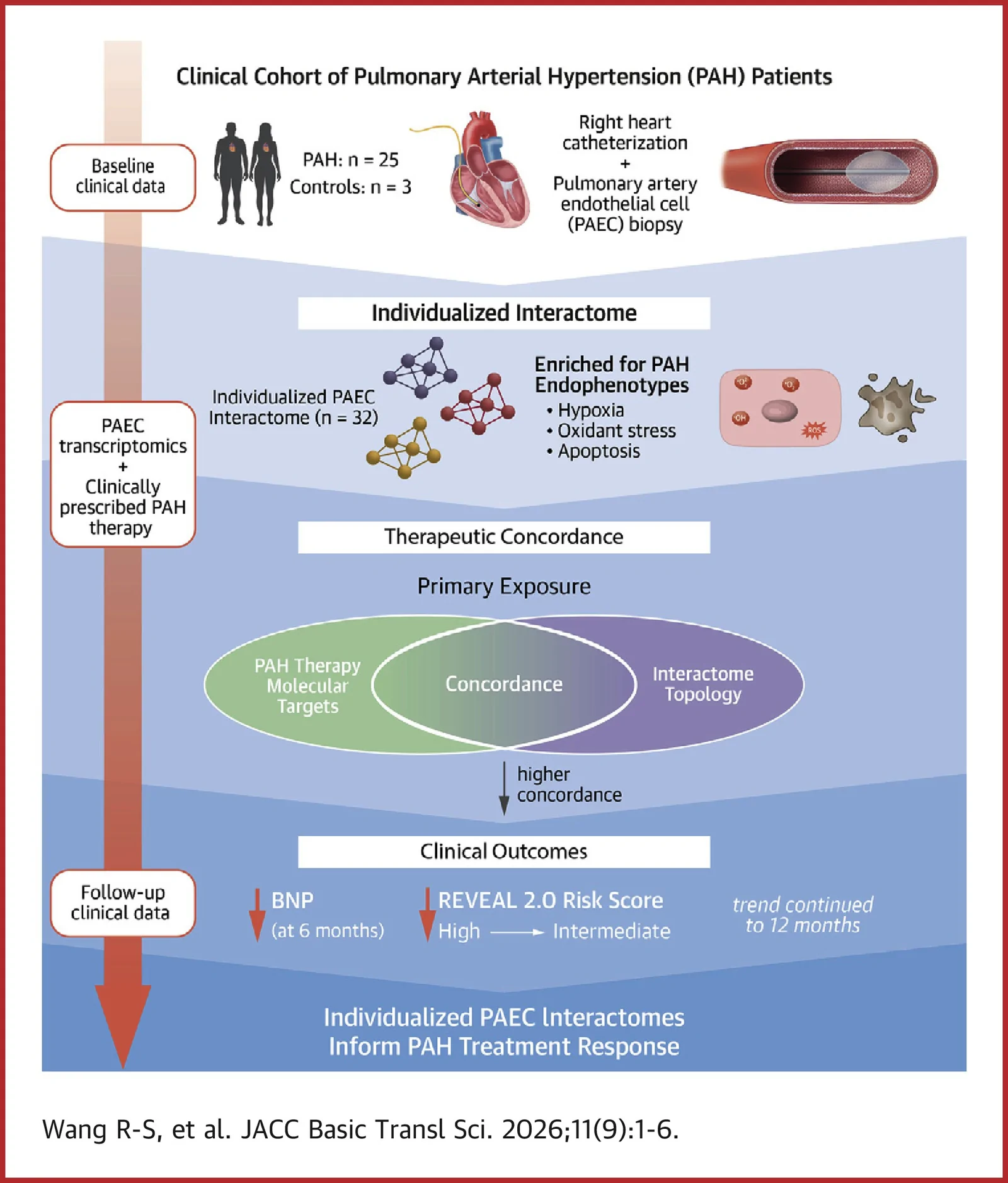

Pulmonary arterial hypertension (PAH) is a highly morbid cardiopulmonary disease characterized by substantial molecular and clinical heterogeneity that drive variable treatment-response patterns observed in clinical trials and real-world practice.^1^, ^2^, ^3^, ^4^ More than 16 medications spanning multiple drug classes are now approved for PAH, and there is increasing emphasis on goal-oriented, combination therapy.^5^ Using patient-specific pathogenetic information to inform drug development, clinical trial design, and treatment selection in clinical practice may improve the efficacy and effectiveness of PAH drugs, respectively, but such strategies are lacking. Progress achieving this goal has been hindered by challenges inherent to sampling affected tissue in PAH and the availability of analytical methods that align individual patient-level pathobiological data to treatment.

A hallmark PAH endophenotype is pulmonary artery endothelial cell (PAEC) dysfunction; ex vivo studies of PAECs from PAH patients validate numerous disease-specific pharmacotherapeutic targets experimentally, but these samples derive largely from end-stage patients at the time of lung explantation.^6^, ^7^, ^8^ We and others have described a minimally invasive “cell biopsy” approach in which PAECs can be collected from the tips of pulmonary artery catheters and propagated in vitro.^9^, ^10^, ^11^, ^12^ Thus, right heart catheterization (RHC) with associated PAEC biopsy provides a direct window into the lung vasculature by sampling a fundamental tissue compartment involved in PAH pathogenesis during a procedure that is central to the diagnosis and longitudinal management of patients.

We recently innovated a network medicine method using transcriptomic data to generate individualized interactomes that emphasize functionally relevant protein-protein interactions (PPIs).^13^ This approach captured patient-specific molecular features (which are otherwise marginalized when using reductionist analytical methods), which could then be used to predict clinical traits in cardiomyopathy patients. In the present study, we established a transcriptome-interactome-systems pharmacology pipeline using PAECs collected at the point of care and implemented this retrospectively using a blinded clinical trial design in silico. We hypothesized that the proportion of therapeutic targets of the prescribed PAH therapy found in each cell biopsy–individualized interactome would be associated with improved clinical outcomes in PAH. Findings from this study are positioned to advance precision medicine by linking patient-level molecular data to treatment selection and, ultimately, therapeutic efficacy and clinical effectiveness.

## Methods

### Overall study design

**Figure 1:**
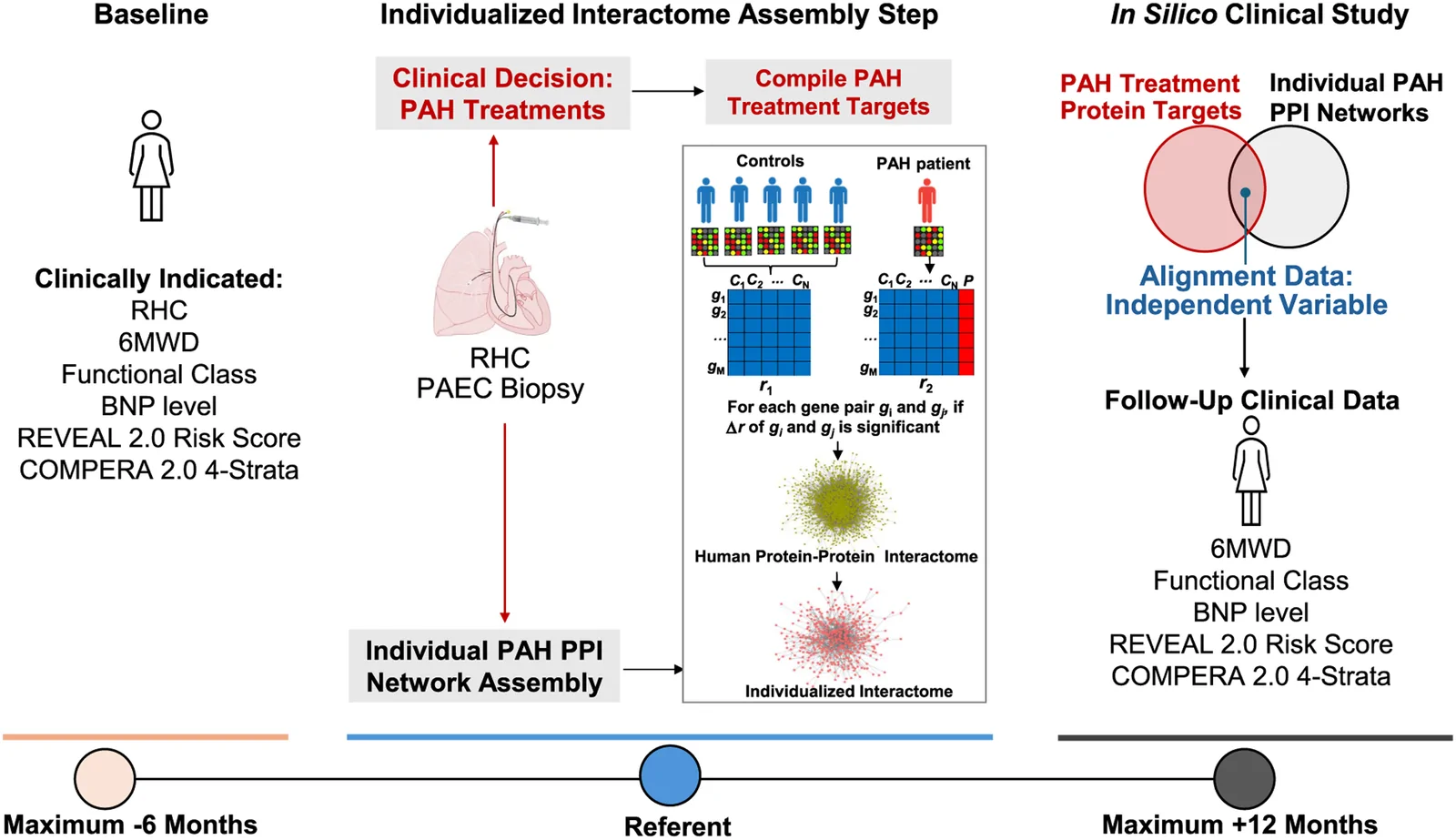
Study Schematic for the In Silico Clinical Trial Clinical data were collected within 6 months before right heart catheterization (RHC) and up to 12 months following RHC. Pulmonary arterial hypertension (PAH) treatments were administered at the time of, or after completion of, the RHC procedure that also included a pulmonary artery endothelial cell (PAEC) biopsy. Following completion of RNA sequencing, an individualized PAH interactome was assembled. The study exposure was the proportion of all protein targets of the PAH pharmacotherapeutic administered to the study patient in their individualized interactome. A similar approach was taken for active and inactive comparators. 6MWD = 6-minute walk distance; BNP = brain natriuretic peptide; COMPERA = Comparative, Prospective Registry of Newly Initiated Therapies for PH; PPI = protein-protein interaction; REVEAL = Registry to Evaluate Early and Long-Term PAH Disease Management.

The overall study design is presented in Figure 1. This was a retrospective analysis of patients undergoing RHC for the routine clinical assessment of dyspnea or PAH monitoring at Rhode Island Hospital (Brown University Health) between 2017 and 2022. All patients were treated according to the usual standard of care (informed by RHC and other results) and at the discretion of their managing PAH clinicians. Clinical data were paired retrospectively with data from patient-specific interactomes assembled from individual PAEC biopsies performed at the time of RHC. A systems pharmacology approach was then used to determine the molecular targets of the exposure, which in this study were U.S. Food and Drug Administration–approved PAH therapeutics (or non-PAH drugs as comparators) prescribed at the time of, or following, RHC. Our goal was to determine if the proportion of therapeutic targets in the individual-specific networks was associated with validated clinical outcome measures assessed as part of routine clinical practice.

### Clinical study design and data collection

Participants were included in this study if they had a clinical diagnosis of PAH using the World Symposium on Pulmonary Hypertension criteria consistent with this diagnosis^14^ (confirmed post hoc by N.S.), underwent successful PAEC biopsy from RHC,^9^^,^^10^ and had ≥1 follow-up clinical assessment after RHC. Baseline clinical data were collected for participants ≤6 months before the date of the study RHC. Post-RHC clinical assessments (eg, 6-minute walk distance [6MWD], risk scores) that occurred up to 12 months after initiation of PAH therapies were included as study endpoints.

### Clinical endpoints

We selected widely used and established clinical metrics in PAH as our study endpoints. The 6MWD is the primary endpoint for almost all phase 3 trials and the basis for drug approval in PAH. The minimal clinically important distance for 6MWD is 30 to 35 m.^15^ Brain natriuretic peptide (BNP) and N-terminal pro-BNP (NT-proBNP) levels are nonspecific markers of right ventricular stretch that are prognostically meaningful in PAH. The cutoff values for BNP of <50, 50 to 199, 200 to 800, and >800 ng/L are ascribed to escalating risk in various multiparameter prognostic models.^5^ Several of these risk assessment scores have been developed for the prognosis and monitoring of PAH and are recommended at diagnosis and frequently thereafter; World Health Organization (WHO) functional class, 6MWD, and BNP or NT-proBNP are the most informative components in all scores. Functional class is one of the most established clinical parameters evaluated in PAH and until recently was the basis of therapeutic escalation.

The REVEAL (Registry to Evaluate Early and Long-Term PAH Disease Management) 2.0 risk calculator has been validated as a prognostic score for predicting 1-year survival.^16^ REVEAL 2.0 incorporates 13 total items, including 6MWD, functional class, and BNP as well as PAH subgroup, demographic information, hospitalizations, echocardiographic findings, vital signs, hemodynamic status, and pulmonary and renal function data. Seven components are required to generate a score, and missing items are assigned a 0 otherwise; risk scores of 0 to 6, 7 and 8, and ≥9 correspond to low, intermediate, and high risk for mortality, respectively. The COMPARA (Comparative, Prospective Registry of Newly Initiated Therapies for PH) 2.0 4-strata risk assessment (1 = low, 2 = intermediate to low, 3 = intermediate high, and 4 = high) for mortality incorporates functional class, 6MWD, and BNP or NT-proBNP. COMPERA 2.0 4-strata risk assessment is recommended to follow PAH patients longitudinally and is the basis for treatment escalation in the most recent consensus guidelines.^5^^,^^17^

Both the clinical data extraction (N.S., H.W., K.C.-F., C.E.V.) and bioinformatics team (R.-S.W., B.A.M.) members worked independently such that PAH treatments, clinical metrics and PPI network results were blinded, respectively. The study was approved by the Institutional Review Board at Brown University Health (IRB#001218).

### Isolation and propagation of PAECs during RHC

Detailed methods and confirmation of PAEC lineage have been published previously.^9^^,^^10^ In brief, following RHC, the pulmonary artery catheter was retracted into the catheter sheath, and both were removed en bloc from the patient. The catheter tip was advanced out of the sheath and placed directly into warm media (37 °C) (EndoGRO, Millipore Sigma) with the balloon deflated and immediately into a heater for travel to the laboratory. The catheter tip with balloon was placed directly into 1 well of a 24-well plate with Attachment Factor Solution (Cell Applications) and washed with fresh media (EndoGRO-VEGF complete media kit) every 2 days. Cells were then seeded into T-25 and T-75 flasks until passaged cells reached confluence over the next 4 to 5 days. Only the clinician conducting the RHC was aware of patient characteristics. The remainder of the study staff members were blinded.

### RNA sequencing and analysis

Transcriptomic analyses were performed on messenger RNA isolated from PAECs at cell passage 3 or 4 for all samples; we have previously demonstrated transcriptome stability across these passages.^2^ Library preparation and RNA sequencing were conducted through Azenta. The same RNA isolation and processing technique was used for all specimens. Specimens were submitted for sequencing in batches and grouped randomly within each batch. Libraries were sequenced using a 2 × 50 bp paired-end rapid run on the Illumina HiSeq2500 platform. Each sample batch was sequenced all at once and distributed equally across sequencing lanes. Technical replicates were not included. All specimens had RNA integrity number values >6 and DV200 > 70%. FastQ files were aligned to the GRCh38 reference sequence using HISAT2,^18^ and gene IDs were resolved using annotateMyIDs.^19^

Across the entire study cohort, there were 3 batches of RNA sequencing data. The first 2 batches included 25,702 genes from 10 unique PAH samples (from 9 PAH participants) and 3 unique nondisease control subjects (individuals referred for RHC for the evaluation of dyspnea but without pulmonary hypertension) and 28,395 genes from 12 individual PAH samples (from 9 PAH participants), respectively. The third batch included 10 additional PAH samples (from 10 PAH participants). We merged the 3 data sets together and removed any transcript with <25 read counts in all the samples to ensure the genes were expressed in either nondisease control subjects or PAH samples. The final data set included 32 PAH samples (from 25 unique PAH participants), 3 nondisease control subjects, and 12,562 genes.

### Construction of individualized PPI interactomes

Sample-specific PPI networks were constructed using methods reported previously with some modifications.^13^^,^^20^ We started with a gene correlation matrix assembled from our nondisease control samples. We calculated Pearson’s correlation coefficient (*r*) for each gene pair across all control samples (n = 3). We then added a single PAH sample and recalculated. If the value of a gene pair was significantly changed (ie, Δ*r* was significant) according to a predefined statistical distribution,^13^^,^^20^ we retained the gene pair. We repeated this calculation for each gene pair. After Bonferroni correction for multiple comparisons and enforcing a threshold on Δ*r* (Δ*r* > 0.5), we mapped significant gene pairs to the human PPI. The human interactome, consolidated from major high-throughput studies, consists of 17,350 proteins (gene products) and 350,950 experimental physical PPIs. The full details of interaction resources used for the most up-to-date version of the human interactome are published.^21^ The gene pairs that had significant Δ*r* and had functional PPIs in the human interactome form the specific network for a given PAH sample. This was repeated for each sample to result in a sample-specific network for each PAEC biopsy. The stability and robustness of the networks was tested by adding synthetic control samples to the control matrix in sensitivity analyses.

### Systems pharmacology analysis

We considered 10 Food and Drug Administration–approved drugs for PAH and 5 calcium-channel blockers and vasodilators that were previously included in the PAH module^21^ and added the investigational drugs dehydroepiandrosterone and sotatercept, given that participants in our center were enrolled in these trials^22^, ^23^, ^24^ (Supplemental Table 1). We extracted the targets of these drugs from DrugBank (Supplemental Table 2) and calculated whether the targets of the drugs were in the individualized interactomes; if so, the number of targets (among all the targets of the drugs) was quantified. The exposure for this study was target-interactome alignment, which was defined as the number of protein targets for a given PAH drug class (eg, phosphodiesterase-5 inhibitors) in an individualized interactome divided by the total number of protein targets in that same drug class (eg, phosphodiesterase-5 inhibitors). In other words, target-interactome alignment was the proportion of drug class targets present in the PAEC biopsy-derived individualized PPI interactome for the therapy administered to a corresponding patient.

### Diuretic, antidepressant, and anxiolytic medications as control comparators

To simulate a placebo-controlled condition in this in silico clinical study, we next chose concomitant medications that are prescribed frequently in PAH but are not considered PAH-targeted therapies, nor are they Food and Drug Administration approved for the treatment of PAH. Specifically, the protein targets of individual diuretic, antidepressant, and anxiolytic medications were compiled from DrugBank (Supplemental Tables 1 and 2). The degree of overlap of the drug targets of these “placebo” drugs to the PAH disease module was calculated to ensure that the “placebo” drugs were not significantly close to the PAH disease module (in essence, the degree to which inactive comparators could be considered active comparators).^21^

We separated diuretic, antidepressant, and anxiolytic medications into 2 groups: those with PAH module overlap and those without. Spironolactone was the only medication with overlap among the prescribed diuretic agents. On the basis of mechanism of action and correlative experimental data, several antidepressant agents targeting serotonin signaling^25^, ^26^, ^27^ (venlafaxine, fluoxetine, quetiapine, escitalopram, desvenlafaxine, mirtazapine, and citalopram) had potential overlap with the PAH module and were therefore analyzed separately (ie, active comparators, grouped as serotonin psychotropic medications) from those that did not overlap with the PAH module (bupropion and benzodiazepines, including clonazepam, alprazolam, and lorazepam) (ie, inactive comparators, grouped as nonserotonin psychotropic medications). The study exposure was analyzed using the same methods as described for PAH therapies.

### Statistical analysis

Continuous data are presented as median (range) and categorical data as frequency (percentage). Associations between continuous variables were estimated using Pearson’s (*r*) or Spearman’s (ρ) correlation coefficient as indicated. Outcomes were modeled as a function of the proportion of protein targets in the sample-specific networks by drug class. Generalized estimating equations were used to account for repeated samples from some participants (25 participants had a total of 7 biological replicates; 1 participant with 5 balloon tips, 3 participants with 2 balloon tips each) and the varying effects of time, given that clinical assessments were not protocolized. An empirical sandwich variance estimator was chosen because of the complexity of the underlying correlation matrix given the patient- and time-based considerations. Regression coefficients (β) are presented with 95% CIs, and *P* values <0.05 were considered to indicate statistical significance. Analyses were conducting using SAS version 9.4 (SAS Institute), and modeling was accomplished using the GLIMMIX procedure (G.B.).

## Results

### Cohort characteristics

The final study cohort included 25 PAH participants and 3 control subjects (individuals who underwent RHC without pulmonary hypertension) contributing 32 and 3 cell biopsy specimens, respectively. The average age of PAH participants was 56 years (range: 30-84 years), and most were women (92%) and White (80%). One participant (4.0%) was not on a PAH-specific treatment at the time of RHC. Most participants were WHO functional class II at the time of RHC, with a median 6MWD of 375 m (range: 150-575 m) and a median REVEAL 2.0 risk score of 10 (range: 4-12). Table 1 includes characteristics of the participants at the time of RHC, and Supplemental Table 3 shows patient subgroups categorized according to PAH treatment pre-RHC and post-RHC.

**Table 1:** Baseline Characteristics of Participants

	Patients With PAH	Control Subjects
Participants	25	3
Balloon tips^a^	32	3
Female sex	23 (92.0)	3 (100.0)
Age, y	56 (30-84)	63 (19-78)
Body mass index, kg/m^2^	32 (16-45)	22 (21-31)
Self-reported race		
White	20 (80.0)	2 (66.7)
Black	2 (8.0)	—
Asian	1 (4.0)	—
Other	2 (8.0)	1 (33.3)
Self-reported Hispanic ethnicity	3 (12.0)	1 (33.3)
Hemodynamic data		
Right atrial pressure, mm Hg	9 (3-19)	7 (1-7)
Mean pulmonary artery pressure, mm Hg	37 (16-69)	18 (16-21)
PAOP, mm Hg	12 (6-17)	10 (8-12)
Cardiac output, L/min	5.3 (1.9-12.9)	5.3 (4.9-5.6)
Pulmonary vascular resistance, Wood units	3.9 (1.0-19.6)	1.8 (1.1-1.9)
PAH etiology		
Idiopathic PAH	7 (28.0)	—
Heritable PAH	3 (12.0)	—
CTD-associated PAH	7 (28.0)	—
Congenital heart disease–associated PAH	2 (8.0)	—
HIV-associated PAH	1 (4.0)	—
Portopulmonary hypertension	1 (4.0)	—
Pulmonary veno-occlusive disease	2 (8.0)	—
Exercise-induced pulmonary hypertension	2 (8.0)	—
Functional class III/IV (n = 6)	2 (33.3)	—
6-min walk distance, m (n = 5)	375 (150-436)	—
Brain natriuretic peptide, pg/mL (n = 5)	359 (34-1,141)	
REVEAL 2.0 risk score (n = 21)	10 (412)	—
PAH treatments		—
None	1 (4.0)	—
Calcium-channel blockers	1 (4.0)	—
PDE5 inhibitors	19 (76.0)	—
Endothelial receptor antagonists	17 (68.0)	—
Prostacyclin pathway	12 (48.0)	—
Investigational	3 (12.0)	
Diuretic agents (n = 21)	20 (95.2)	—
Psychotropics, serotonin	9 (36.0)	—
Psychotropics, nonserotonin	6 (24.0)	—

Values are n, n (%), or median (range). PAH was defined as mPAP > 20 mm Hg, PAOP < 15 mmHg, and pulmonary vascular resistance > 3.0 Wood units at diagnosis. Hemodynamic data may fall outside of these definitions at time of right heart catheterization and cell biopsy.

CO = cardiac output; CTD = connective tissue disease; mPAP = mean pulmonary artery pressure; PAH = pulmonary arterial hypertension; PAOP = pulmonary artery occlusion pressure; PDE5 = phosphodiesterase-5; REVEAL = Registry to Evaluate Early and Long-Term PAH Disease Management.

a Twenty-five participants had a total of 7 biological replicates (1 participant with 5 balloon tips, 3 participants with 2 balloon tips). Exercise-induced pulmonary hypertension was defined as resting mPAP < 20 mm Hg and with exercise, ΔmPAP/ΔCO (by thermodilution) > 3 mm Hg/L/min.

### Transcriptomic profile of participants

The PAEC RNA sequencing transcriptomic data set, after filtering, included 32 PAH samples (from 25 unique PAH patients), 3 nondisease control samples, and 12,562 genes. The multidimensional scaling plot of the data set shows that the variance in gene expression explained by the first and second principal component vectors was 27% and 11%, respectively, with separation between the nondisease control and PAH groups but also substantial separation within the PAH group (Supplemental Figure 1A). The coefficient of variation range for the expression of genes among PAH samples was 33% to 545%, with 6,545 (52%) genes having coefficients of variation ≥80% (Supplemental Figure 1B). The average correlation coefficient for replicates in nondisease control samples was 0.91, whereas the average correlation coefficient of replicates in PAH samples was 0.81 (Supplemental Figure 2). These collective data suggest important pathobiological variability across the PAH samples and are consistent with the clinical heterogeneity observed in this (and many other) study populations.^1^^,^^28^^,^^29^

### Individualized interactomes

The individualized PAH interactomes varied widely in topology (median number of nodes 5,954; range: 2,074-9,569) and complexity (median number of edges 8,494; range: 2,097-24,877) (Table 2, Supplemental Figure 3). The messenger RNA expression pattern of PAH pharmacotherapeutic targets across individualized interactomes is presented in Supplemental Figure 4. We used the overlap coefficient, a similarity measure that measures the overlap between 2 finite sets, to evaluate the node similarity and edge similarity of 2 individualized PAH interactomes.^13^ The pairwise node similarity of individualized interactomes displays a modular pattern, with an average of 0.77 (range: 0.46-0.93). The edge similarity of individualized interactomes varied substantially, from 0.12 to 0.57 (Supplemental Figure 5). A much greater overlap for nodes was observed compared with edges across the interactomes. The uniqueness of sample-specific interactomes (ie, the nodes and edges that do not appear in any other networks) ranged from 0 to 37 nodes (median 3) and from 8 to 4,119 edges (median 219). This finding emphasizes that it is the wiring diagrams (PPIs), rather than the proteins per se, that distinguished patients in this study. Topological analyses of the individualized PAH interactomes show that although all these networks have similar density, they have a wide range of network diameter, characteristic path length, and size of largest connected components (Supplemental Table 4).

**Table 2:** The Profile of Individualized Interactomes Stratified by Participant and Pulmonary Artery Endothelial Cell Sample and After Adjustment for Multiple Comparisons

		Adjusted *P* < 0.05, |Δ*r*| > 0.5	
Participant^a^	EC Sample	Number of Proteins (Nodes)	Number of Interactions
P005E	EC1	7,619	13,914
P014	EC2	7,989	16,492
P010B	EC3	8,027	16,961
P001	EC4	6,594	10,091
P004	EC5	7,257	14,259
P007	EC6	7,446	13,550
P008	EC7	7,654	14,228
P010A	EC8	7,347	12,724
P011	EC9	6,704	10,756
P012	EC10	6,642	10,347
P017	EC11	2,753	2,950
P016	EC12	4,027	4,813
P015	EC13	2,313	2,369
P005D	EC14	3,874	4,354
P002	EC15	3,029	3,285
P003	EC16	3,119	3,448
P005A	EC17	2,737	2,914
P005B	EC18	2,905	3,078
P005C	EC19	2,609	2,763
P006	EC20	2,074	2,097
P009	EC21	2,428	2,628
P013	EC22	3,350	3,939
P012B	EC23	7,685	14,396
P018	EC24	7,231	12,913
P019	EC25	5,135	6,632
P020	EC26	5,313	6,896
P021	EC27	7,254	12,650
P022	EC28	8,828	20,023
P008B	EC29	4,820	5,972
P023	EC30	9,569	24,877
P024	EC31	7,855	15,180
P025	EC32	5,215	6,711

Participant sample numbers represent individual patients.

Δr = change in Pearson’s correlation coefficient; EC = endothelial cell.

a P = patient, the 3-digit number is the sample identification, and the single-letter code represent a unique pulmonary artery endothelial cell sample.

We next performed a subgroup-level analysis to determine if unique molecular signatures aligned with clinical phenotypes. On the basis of the multidimensional scaling and edge similarity data, 3 distinct subclusters were identified by using hierarchical clustering (Supplemental Table 5, Supplemental Figure 6), reinforcing the utility of network medicine for segregating molecular endotypes among patients that share the same diagnosis of PAH. Moreover, across these clusters there were no clear clinical phenotypic differences.

The principal features of PAH include a variety of phenotypes, such as fibrosis, inflammation, levels of oxidative stress, hypertrophy, and others.^30^ To demonstrate that the interactomes can capture individual-level pathobiological differences and phenotypic heterogeneity, we conducted an endophenotype enrichment analysis. Of 75 endophenotypes, 34 endophenotypes were enriched significantly (*P* < 0.05 adjusted using the Benjamini-Hochberg procedure) in at least 1 sample. A heatmap showing the statistically significant associations (−log_10_*P*) is shown in Supplemental Figure 7. All of the individualized PAH interactomes were enriched significantly with genes associated with hypoxia, oxidative stress, and apoptosis, and the majority were enriched significantly with DNA damage, angiogenesis, cell proliferation, and immune response genes. There was greater variation across PAH patients in enrichment for genes associated with fibrosis, thrombosis, and inflammation. The results demonstrate the endophenotypic heterogeneity of these patients and the capability of patient-specific networks to capture individual-level differences.

### PAH therapeutic target-interactome alignment and PAH clinical endpoints

**Figure 2:**
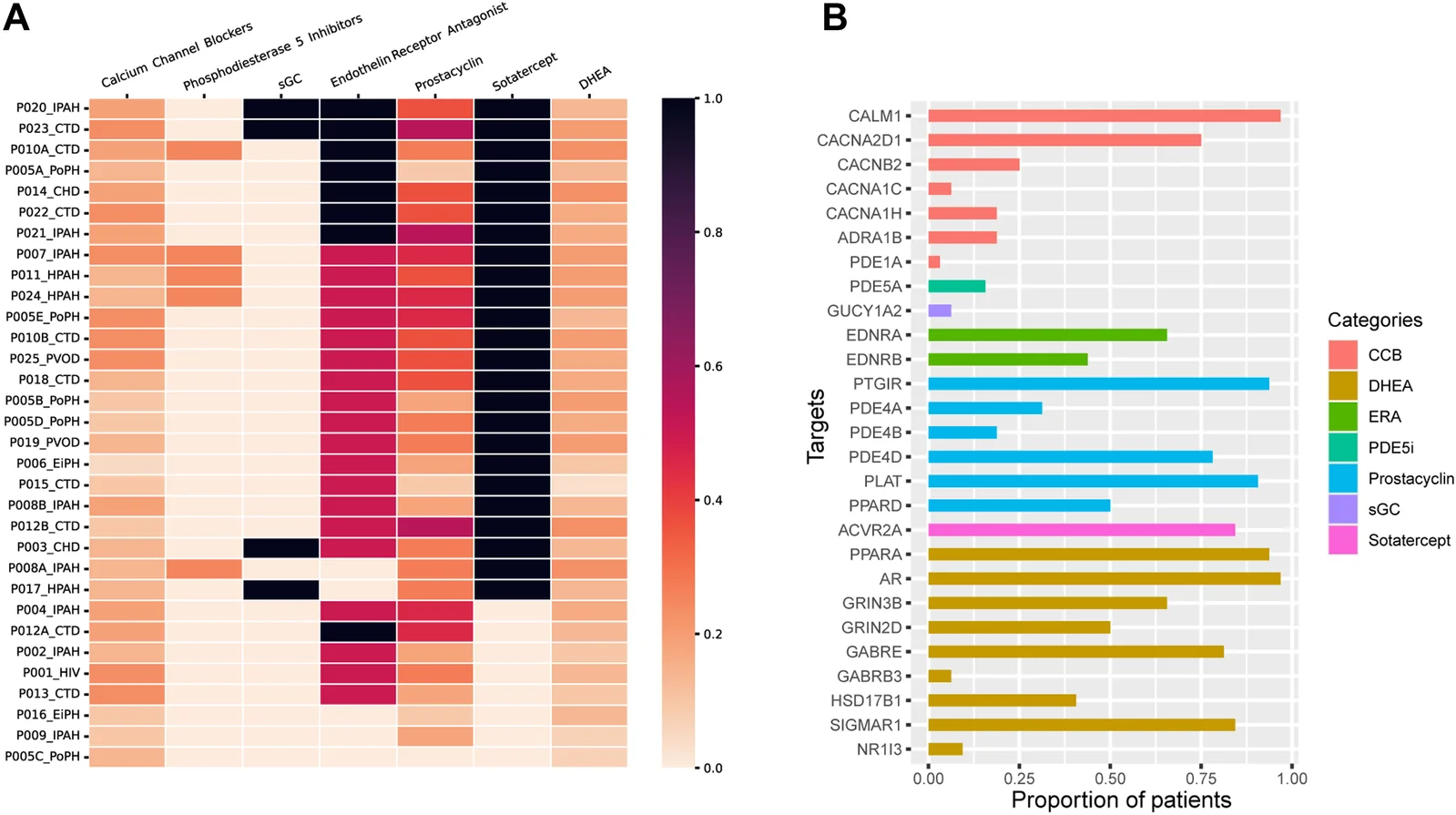
Heterogeneity of PAH Pharmacotherapeutic Targets in the Individualized PAH Interactomes (A) Pulmonary artery endothelial cell biopsies collected from pulmonary arterial hypertension (PAH) patients at the point of care and their transcriptomic data were used to assemble individualized interactomes. Heatmap showing the proportion of protein targets within a drug class in each individualized PAH interactome. Each row represents an individual participant. Each column represents a drug class. Each cell indicates the proportion of targets expressed by an individual patient. Black, 100% targets expressed; purple, 60% targets expressed; red, 40% targets expressed; orange, 20% targets expressed; peach, 0% targets expressed. (B) Proportion of participants who expressed each target (y-axis label). Color-coded legend: red, calcium-channel blocker (CCB); orange, dehydroepiandrosterone (DHEA); green, endothelin receptor antagonist (ERA); teal, phosphodiesterase 5 inhibitor (PDE5i); blue, prostacyclin; purple, stimulator of soluble guanylate cyclase (sGC); pink, sotatercept. CHD = coronary heart disease; CTD = connective tissue disease; EiPH = exercise-induced pulmonary hypertension; HPAH = heritable pulmonary arterial hypertension; IPAH = idiopathic pulmonary arterial hypertension; PoPH = portopulmonary hypertension; PVOD = pulmonary veno-occlusive disease.

We identified 31 PAH, 3 diuretic, 9 serotonin psychotropic, and 2 nonserotonin psychotropic pharmacotherapeutic targets in individualized interactomes. A heatmap showing the proportion of targets within a drug class present in the individualized interactome is provided in Figure 2A. The quantitative rank-order summary of patient-specific PAH and comparator pharmacotherapeutic protein targets is provided in Supplemental Tables 6 to 10 and indicates a non-normal distribution of targets in individualized interactomes for PAH and comparator drugs. Notably, there was wide variation in the proportion of individuals expressing targets for PAH-specific classes (Figure 2B).

**Figure 3:**
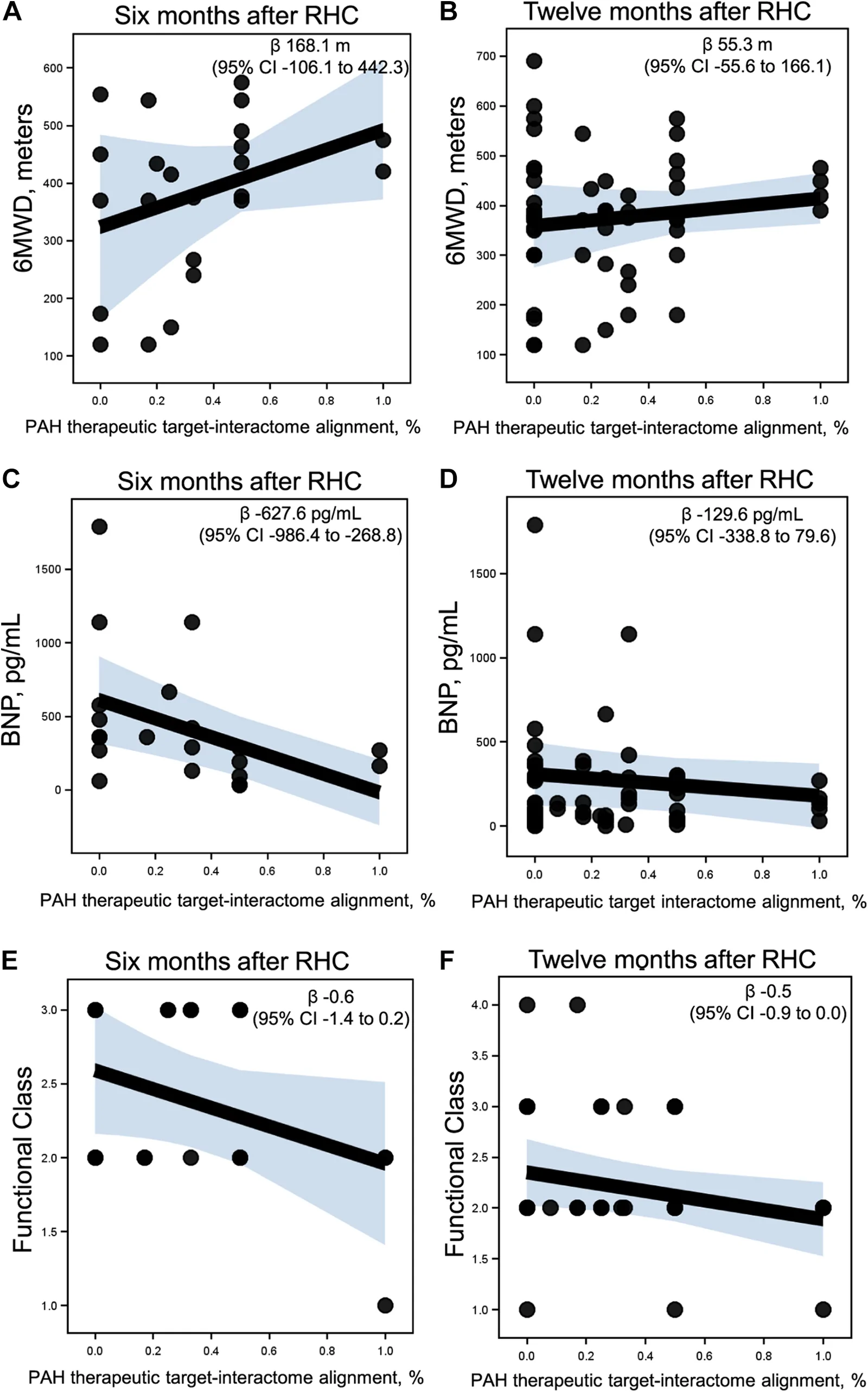
In Silico Clinical Trial Results Association as determined using linear regression (β = coefficient) between PAH therapeutic target-interactome alignment and clinical outcomes. 6MWD (A, B), BNP level (C, D), and functional class (E, F) at 6 and 12 months after RHC. Abbreviations as in Figure 1.

Greater PAH pharmacotherapeutic target-interactome alignment was associated with improvements in multiple PAH clinical endpoints. For example, complete alignment was associated with increases in 6MWD of approximately 160 m at 6 months and 55 m at 12 months, although the overall associations did not reach statistical significance, likely because of limited sample size (eg, 12 months: β = 55.3 m; 95% CI: −55.6 to 166.1 m; *P* = 0.20) (Figures 3A and 3B). Similarly, we observed a transition in BNP levels from elevated at baseline to within the normal range that corresponded to an absolute decrease of 627 pg/mL (95% CI: −986.4 to −268.8 pg/mL; *P* = 0.003) at 6 months (Figure 3C), an association that persisted but was not significant at 12 months (β = −129.6; 95% CI: −338.8 to 79.6; *P* = 0.22) (Figure 3D). Therapeutic class target-interactome alignment demonstrated a nonsignificant association with WHO functional class improvement at 6 months (β = −0.63; *P* = 0.11) and 12 months (β = −0.46; *P* = 0.06) (Figures 3E and 3F).

**Figure 4:**
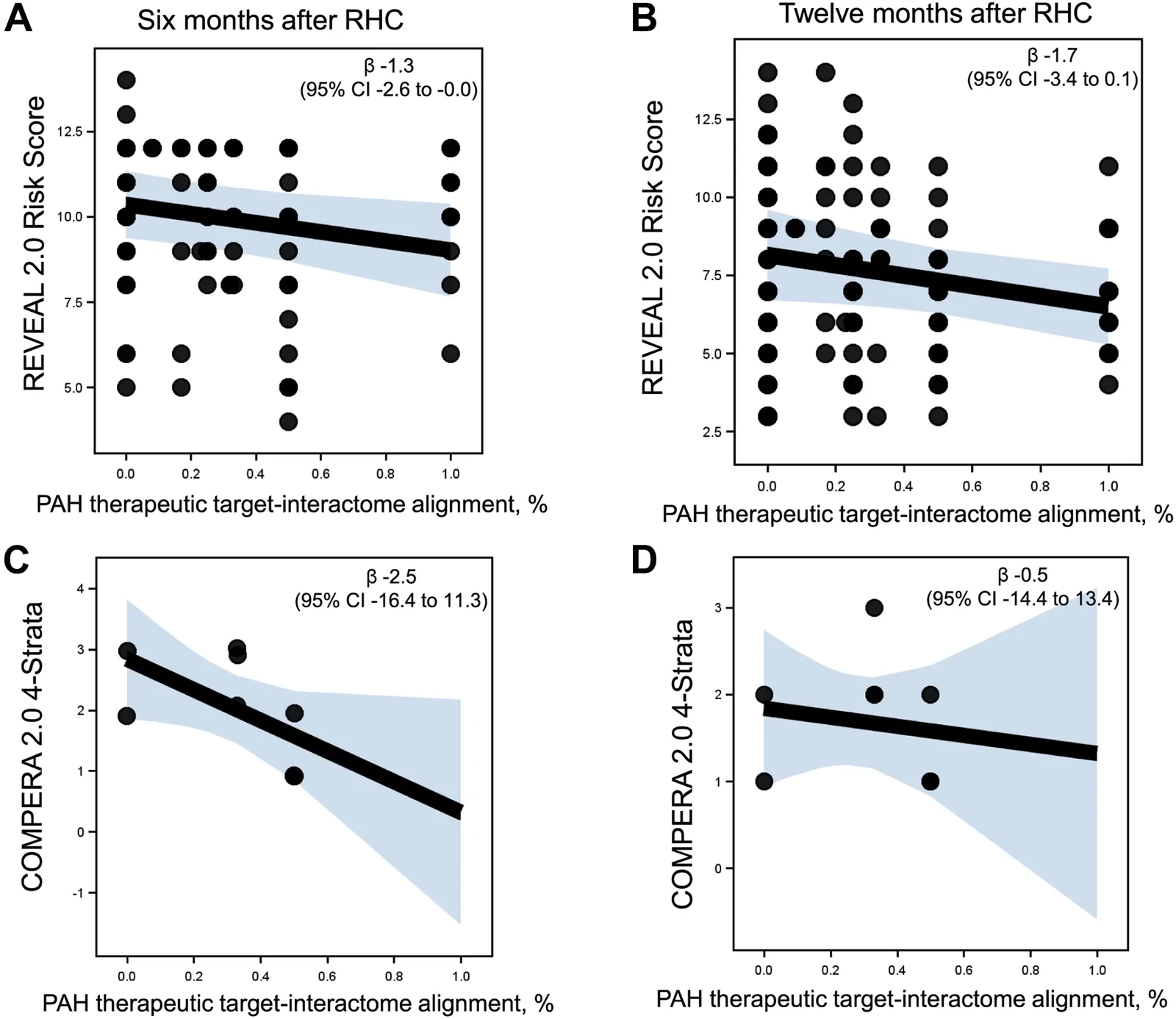
In Silico Clinical Trial Results Association as determined using linear regression (β = coefficient) between PAH therapeutic target-interactome alignment and clinical outcomes. Improvement in the REVEAL 2.0 risk score (A and B) and the COMPERA 2.0 4-strata risk assessment (C and D) at 6 and 12 months after RHC, respectively. Abbreviations as in Figure 1.

Greater therapeutic target-interactome alignment was associated with a significant improvement in REVEAL 2.0 risk score at 6 months (β = −1.3; 95% CI: −2.6 to −0.043; *P* = 0.04) (Figure 4A) and 12 months (β = −1.7; 95% CI: −3.4 to 0.07; *P* = 0.059) (Figure 4B), ranging generally from intermediate risk (7 or 8) to high risk (≥9) for no alignment to intermediate or low risk (≤8) for mortality with complete alignment. A nonsignificant association was found between greater therapeutic class target-treatment concordance and lower COMPERA 2.0 4-strata risk assessments at 6 months (β = −2.5; 95% CI: −16.4 to 11.3; *P* = 0.26) (Figure 4C), a relationship not observed at 12 months (Figure 4D).

### Pharmacotherapeutic comparators, target-interactome alignment, and PAH endpoints

Among the 25 PAH participants in this study, 20 (80.0%), 8 (32.0%), and 7 (28.0%) were prescribed diuretic medications and drugs included in the serotonin psychotropic and nonserotonin psychotropic groups, respectively, at the time of RHC. Spironolactone was the only diuretic agent for which ≥1 drug-specific molecular target was found to overlap in the PAH module but was prescribed too infrequently (n = 1) in this study population for further analysis. Among participants prescribed diuretic agents exclusive of spironolactone at the time of RHC, there was no relationship identified between therapeutic target-interactome alignment and 6MWD, BNP level, or WHO functional class at 6 or 12 months (data not shown). Similarly, no associations were identified between diuretic target-interactome alignment and risk scores at 6 or 12 months (data not shown).

Among those prescribed serotonin psychotropic drugs, increasing alignment was associated with worsening REVEAL 2.0 scores. As concordance increased, REVEAL 2.0 risk scores worsened from low to high risk for mortality at 6 months (β = 2.7; *P* = 0.034), a relationship that was attenuated at 12 months (β = 1.9; *P* = 0.18) (Supplemental Figure 8). Conversely, participants who were prescribed nonserotonin psychotropic drugs at the time of RHC and had target alignment were significantly more likely to have lower REVEAL 2.0 risk scores at 6 months, such that alignment in gene targets was associated with a change from high risk (≥9) to intermediate risk (7 or 8) (β = −2.5; *P* = 0.050), a relationship that was not observed at 12 months (β = −0.8; *P* = 0.59). Gene target–nonserotonin psychotropic alignment had no detectable relationship with other PAH endpoints.

### Temporal changes in networks over the disease course

**Figure 5:**
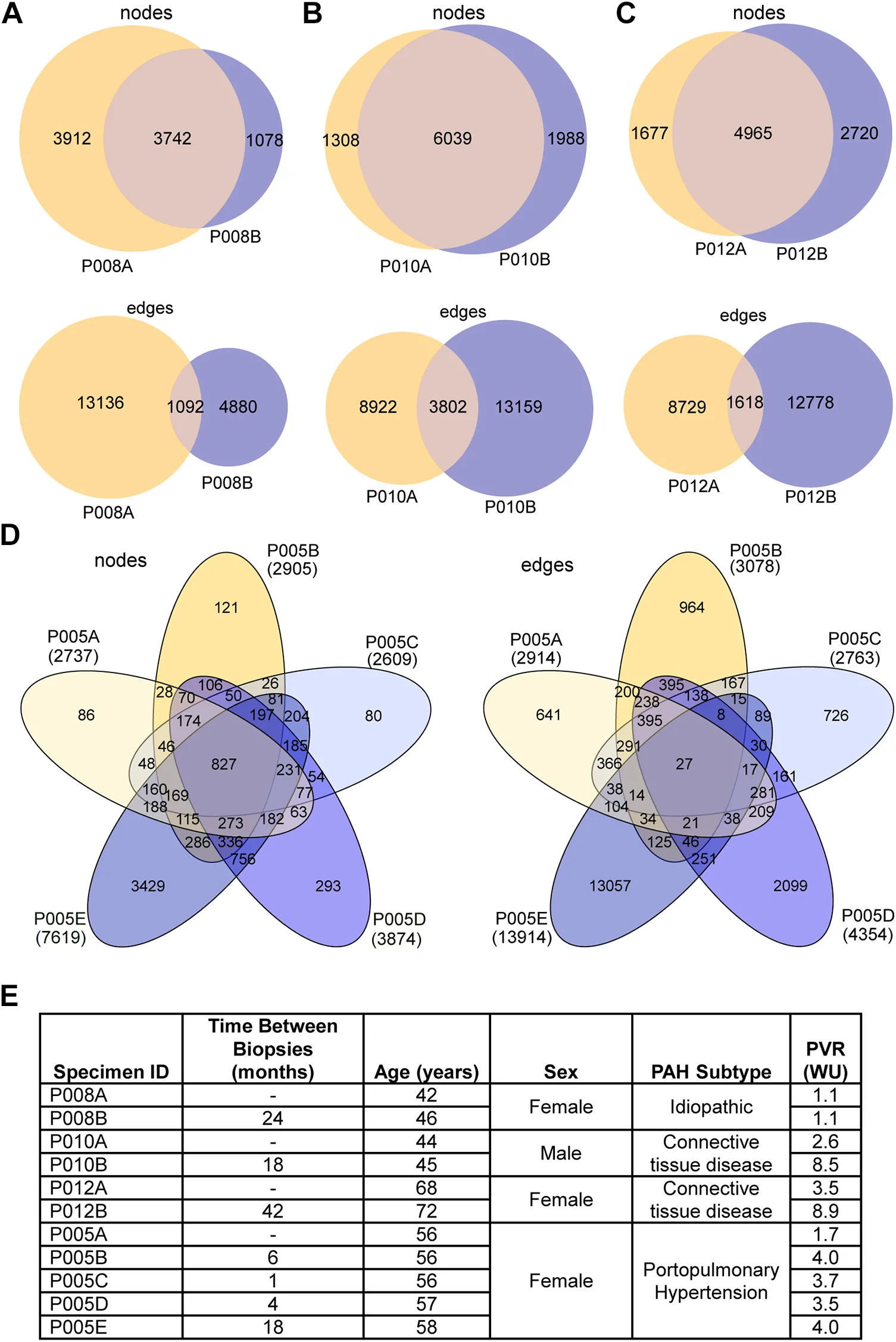
Temporal Changes in the Profile of Individualized Interactomes for 4 PAH Participants in This Study (A-D) Individualized interactomes for 4 pulmonary arterial hypertension (PAH) participants. (E) Select clinical data. PVR = pulmonary vascular resistance; WU = Wood units.

**Figure 6:**
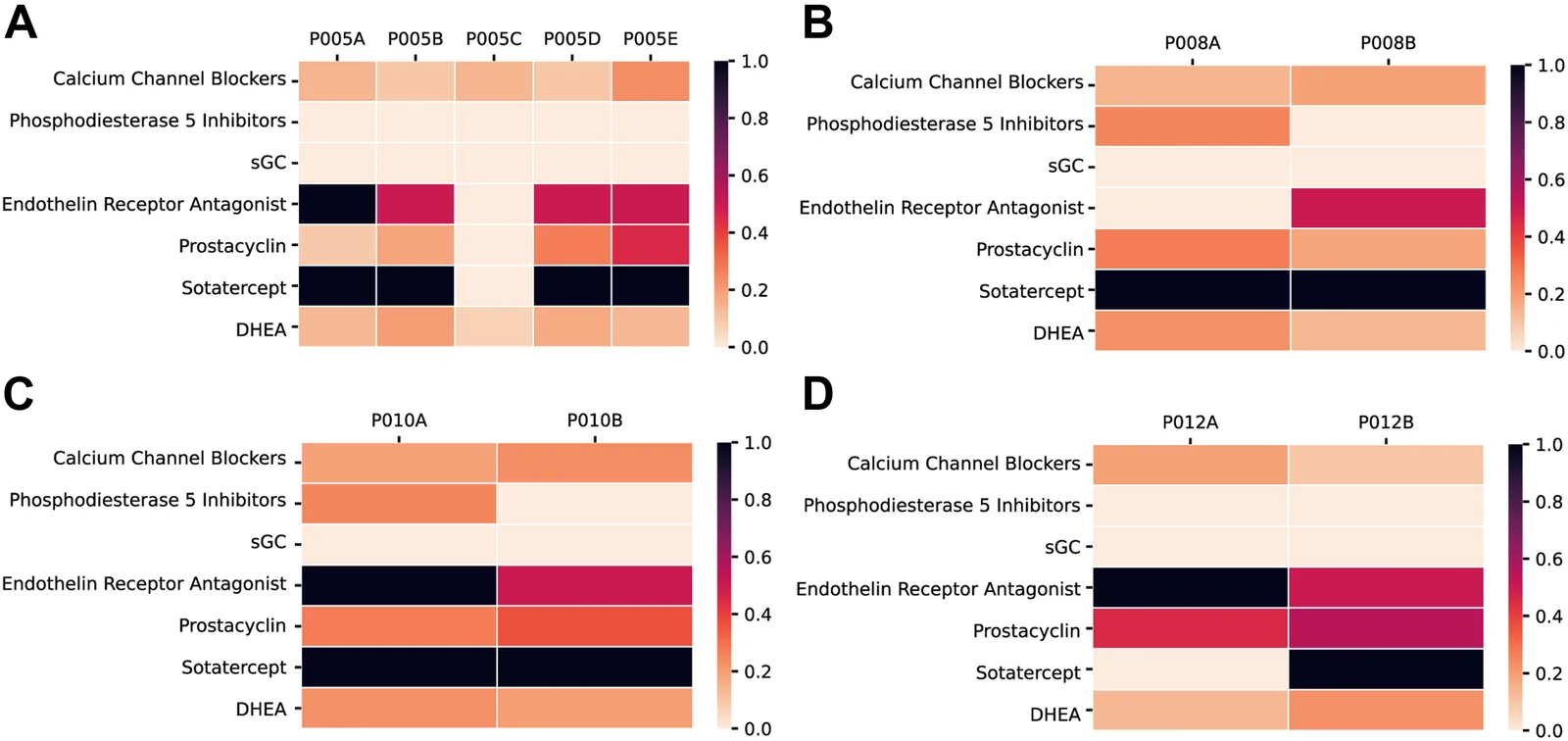
Changes in Pharmacotherapeutic Target-Interactome Alignment (by Drug Class) Across Biological Replicates (A) A 56-year-old woman with portopulmonary hypertension had progressive disease with escalating therapy over serial RHCs (P005A-P005D; PVR 1.7, 4.0, 3.7, and 3.5 WU respectively). A final sample was obtained after liver transplantation, at which time the patient was no longer taking pulmonary vasodilators and hemodynamic status had improved (P005E; PVR 4.0 WU). (B) A 42-year-old woman with a known diagnosis of idiopathic PAH on a PDE5i and an ERA experienced persistent dyspnea and right ventricular dysfunction on echocardiography (P008A; PVR 4.8 WU). She was enrolled in STELLAR (A Study of Sotatercept for the Treatment of Pulmonary Arterial Hypertension [MK-7962-003/A011-11]), a phase 3, multicenter, double-blind, randomized, placebo-controlled trial of sotatercept in which the patient was randomized to the treatment arm (NCT04576988). Repeat RHC was performed as part of the study, at which time she reported diminished dyspnea (P008B; PVR 7.7 WU). (C) A 44-year-old man with systemic sclerosis–associated PAH experienced clinical worsening (P010B; PVR 8.9 WU), ultimately requiring lung transplantation. Pathologic examination of the explant demonstrated pulmonary venule occlusion. (D) A 68-year-old woman with limited systemic sclerosis and progressive dyspnea was diagnosed with PAH (P012A; PVR 3.5 WU) and had progressive dyspnea requiring follow-up RHC (P012B; PVR 8.9 WU). Each row represents a drug class. Columns represent serial pulmonary artery endothelial cell sampling via RHC. Data are expressed as the proportion of therapeutic targets (by drug class) in the individualized interactomes. Each cell indicates the proportion of targets expressed at the time of RHC. Black, 100% targets expressed; peach, 0% targets expressed. Abbreviations as in Figures 1, 2, and 5.

We monitored for temporal changes in individualized interactomes from repeated biopsies in 4 PAH patients (n = 5 biopsies for P005, n = 2 biopsies each for P008, P010, and P012). Substantial network rewiring was observed for P008, in which 77% of nodes and only 18% of edges were shared across time points. In contrast, for P010, the overall network size increased progressively across each biopsy sample, although only 827 proteins and 27 edges shared all 5 interactomes (Figure 5). Overall, the proportion of drug class targets in the interactomes varied within and between patients for whom repeated samples were available for analysis (Figure 6).

## Discussion

In this study, a novel network medicine approach integrating transcriptomics from PAEC biopsies acquired from PAH patients at the point of care with the human protein-protein interactome was used to link individualized treatment selection to clinically relevant endpoints. In doing so, this work progresses precision medicine through 3 key advances. First, we established an analytical pipeline integrating functional PPIs, derived from patient cells ex vivo and modeled in silico, with systems pharmacology which was associated with patient-level therapeutic responses. Second, we demonstrated the potential for individualized interactomes to harbor clinically actionable data, which we tested with a novel study design pairing patient-specific pathobiological readouts to drug effects. Third, findings from this study emphasize the broad translational potential of cell biopsies collected during routine clinical practice despite (or perhaps as a function of) variability across samples. Taken together, these findings provide a basis for further prospective studies scaling patient-specific treatment regimens on the basis of individualized therapeutic response to a standard-of-care approach in PAH, with implications for other complex diseases characterized by both diverse pathogenetic causes and variable outcomes.

Greater appreciation of the clinical and molecular diversity underpinning pulmonary vascular diseases has stimulated efforts to reclassify PAH patients on the basis of deep phenotyping. Distinct endophenotypes determined by peripheral blood signatures (ie, inflammation, monogenic variants, others) are associated with differences in PAH severity and outcome.^28^^,^^31^, ^32^, ^33^, ^34^, ^35^, ^36^ However, approaches used in these prior studies tended to use analytical methods that may overemphasize single molecular events or bystander (rather than functional) biomarkers to explain pathogenicity and have not been individualized or used to inform treatment selection successfully. Here, we used (valid) functional PPIs to assemble biopsy-specific interactomes, characterize pathobiological patterns between patients, and test associations with PAH pharmacotherapeutic response. We observed general agreement in network topology across patients when considering proteins (nodes), but substantial variability ranging from near total to near absent overlap between patients (including in repeated samples from the same patients) when focusing on functional PPIs. These findings imply that phenotypic heterogeneity across patients (and disease course) with PAH hinges on molecular connectivity detectable in affected tissue (ie, PAECs), rather than simple expression profiles. This has important implications for treatment in PAH, as uncoupling between transcript quantity and pathogenicity is common in complex diseases,^37^ whereas PPIs implicated in individualized interactome connectivity itself appeared in this study to be a measure of functional importance.

Despite acknowledgment of the need for deep phenotyping and precision-based treatment approaches in PAH, especially given the evolving treatment landscape, challenges remain in operationalizing these concepts. Because PAH remains a highly morbid disease, guidelines have understandably emphasized initial combination therapy and treatment escalation to improve outcomes.^5^ Treatment response heterogeneity has been identified in PAH clinical trials and registries based on patient comorbidities,^2^^,^^3^ which could inform prognostic enrichment, but an equally important goal would be to enhance drug development and clinical trial efficiency via predictive enrichment (eg, targeting patient-level molecular signatures). At the bedside, clinician selection of precise PAH therapeutic regimens would ideally improve outcomes and health-related quality of life while minimizing side effects, burden, and cost. This proof-of-concept in silico trial, which derives individualized networks from cell biopsies of the affected tissue compartment and links network topology to associated clinical metrics, is the first step toward a point-of-care process for therapeutic efficacy and effectiveness screening and personalized medicine in PAH. Optimizing the analytical pipeline by scaling the cell biopsy approach prospectively and with standardized endpoints, implementing real-time transcriptomics, and semiautomating the bioinformatic pipeline is necessary to validate the applicability and feasibility of the methods.

Gene target–PAH therapeutic class alignment was associated with improvement in multiple, directionally consistent and important PAH endpoints used for drug approval or treatment escalation in clinical guidelines. Some of these relationships persisted for up to 1 year after introduction of therapy. The effect estimate observed for 6MWD at 6 months—more than 160 m—well surpassed the minimally clinically important difference of approximately 33 m for 6MWD in PAH.^15^ Gene target–PAH therapeutic class alignment was also associated with normalization of BNP levels to low categories of PAH risk.^5^ Estimates for REVEAL 2.0 risk and COMPERA 2.0 risk assessments were also decreased, which has been associated with improved survival and a reduction in important clinical events including lung transplant and hospitalizations.^16^^,^^38^ These signals were detected despite a relatively small sample size, the noncontrolled study design and background therapies, data missingness (addressed in the following discussion), and analyses at the therapeutic class (not specific drug) level, which would all serve to increase the signal to noise ratio and bias our results to the null.

Serotonin synthesis and signaling is implicated in PAH pathobiology.^25^^,^^27^ Many observational studies have demonstrated relationships between antidepressants, specifically selective serotonin reuptake inhibitors, and risk in variable forms of pulmonary vascular disease.^26^ More recently, rodatristat ethyl was developed to enhance serotonin synthesis by peripheral inhibition of tryptophan hydroxylase 1 and tested in a phase 2b study terminated for safety.^39^ We observed similar relationships in the present study, in which psychotropic medications targeting serotonin signaling were possibly associated with worse PAH metrics. Confirmation of overlap of these drugs in the PAH module, detection of related gene targets in our cell biopsy samples, and linking of greater target engagement with PAH outcomes in a direction supported by empirical research provides key measure of validity of to our approach as well as insight into molecular phenotypes in PAH patients per se that may be informative in clinical studies involving these drugs.

No relationships were detected between PPI alignment and diuretic medications outside of the PAH module (a pure “placebo” condition) and PAH endpoints, as hypothesized given that no direct molecular targets were found (nor anticipated) in the PAH module. Target alignment with nonserotonin psychotropic medications was associated with improved REVEAL 2.0 risk scores but had no associations with 6MWD, BNP, or functional class. As these treatments had no overlap in the PAH module (and therefore would be unlikely to target biologic pathways relevant to pulmonary vascular disease in the study participants), we speculate this could be due to confounding by indication. In other words, patients who were experiencing progressive disease (ie, high REVEAL 2.0 risk scores at the time of cell biopsy) were more likely to be prescribed psychotropic medications, given the known high concurrent rate of anxiety and depression with PAH,^40^^,^^41^ with subsequent reduction in REVEAL scores over time with PAH treatment.

### Study limitations

Although findings from this study advance personalized therapeutic selection using clinically relevant evidence, prospective research with protocolized study design and clinical assessments (eg, a randomized clinical trial) is needed to clarify the translatability of our methodology (and derivative findings) to clinical practice. These samples were primarily collected over the course of the COVID-19 pandemic, and as such there was more missing clinical data after treatments were introduced than would be expected, and we cannot fully know the mechanisms of missingness (at random, not at random).^42^ The overall study population size was modest, and the sizes of some subgroups (eg, patients prescribed spironolactone) limited statistical analyses. Although the number of control samples available for assembling the interactomes was small, we confirmed robustness and stability of the interactomes using additional synthetic controls (Supplemental Figure 9). Men were underrepresented in this study, and therefore, sex imbalance is an important potential confounder of our results. In the case of REVEAL 2.0 risk scores, some missing values were assigned a value of 0 albeit using a validated approach.^16^ As we do not routinely perform RHC at standard intervals for all patients clinically, selection or referral bias may have influenced our results. Baseline PAH severity as well as the number of prevalent vs incident (ie, treatment-naive) participants included may also have influenced our observations. Additional sensitivity analyses in which we controlled for baseline clinical measures did not change the results. Finally, although drug classes differ in the number of annotated targets, there was no correlation between the number of unique targets per PAH drug class and target-interactome alignment (and our analysis looked at proportion, not absolute number, of targets) (Supplemental Figure 10), arguing against bias introduced because a given PAH drug class had greater target enrichment. We did not routinely collect data on health-related quality of life or therapeutic burden, although this will be important in future precision-based efforts.

## Conclusions

Pulmonary endothelial biopsy data collected at the point of care in PAH and analyzed using a network medicine-systems pharmacology pipeline harbors clinically actionable information for PAH patients. Findings from this study advance precision medicine into a framework in which individualized clinical decision-making is possible and warrants future prospective in silico studies validating bioinformatics-based approaches to treatment selection and clinical trial design in PAH, as well as other complex diseases defined by pathophenotypic heterogeneity.

Perspectives **COMPETENCY IN MEDICAL KNOWLEDGE:** Selecting therapies in patients with PAH is based currently on risk assessed by clinical parameters. By contrast, key molecular features that drive the pathophenotype in individual patients have been proposed as potentially helpful in clinical decision-making. To align the pathobiology of PAH with therapeutic selection requires access to affected tissue and a methodology that individualizes the PAH molecular signature while focusing on functionally relevant pathways. This study advances precision medicine by showing that biopsies collected at the point of care during a clinically indicated test support a network medicine approach for individualized therapeutic selection in PAH.

**TRANSLATIONAL OUTLOOK:** This study has direct translational relevance to medicine by bridging a long-standing barrier to clinically actionable molecular data in heterogeneous and complex diseases.

## Funding Support and Author Disclosures

This work was completed with support from National Institutes of Health grants R01-HL141268 (to Dr Ventetuolo), R01-HL174007 (to Dr Ventetuolo), P20-GM103652 (to Drs Harrington and Ventetuolo), T32-HL134625 (to Drs Ventetuolo, Singh and Cox-Flaherty), R01-HL139613 (to Dr Maron), R01-HL153502 (to Dr Maron), and R01-HL155096 (to Dr Maron). Dr C.J.M. has received personal fees from Merck and Regeneron, outside of the submitted work. Dr Ventetuolo has received personal fees from Merck, Janssen Pharmaceuticals, Pulmovant, and Regeneron, outside of the submitted work; and has received fees for the conduct of clinical trials, paid to her institution, from Merck, Pulmovant, Tenax, Gossamer, and United Therapeutics. Drs Ventetuolo and Maron report patent applications BM-2024-052 and 15024-398PC0 (pending). Dr Maron has patent PCT/US2019/059890 (pending), patent 9,605,047 (issued), patent PCT/US2020/066886 (pending), and patent BWH 2023-152-29618-0438P02 (pending); has received funding from the National Institutes of Health; and is an investigator at the University of Maryland Institute for Health Computing, which is supported by funding from Montgomery County, Maryland, and The University of Maryland Strategic Partnership (MPowering the State, a formal collaboration between the University of Maryland, College Park, and the University of Maryland, Baltimore). All other authors have reported that they have no relationships relevant to the contents of this paper to disclose.
