# Supplementary material for: Individualized Interactomes From Pulmonary Arterial Hypertension Cell Biopsies Inform Pharmacotherapeutic Response
Source: JACC Basic Transl Sci. 2026 Aug 18;11(9):101655. doi: 10.1016/j.jacbts.2026.101655 (PMC13519260; doi:10.1016/j.jacbts.2026.101655)
Supplement: Supplementary file 1 [file mmc1.docx]

**Individualized Interactomes from Pulmonary Arterial Hypertension Cell Biopsies**

**Inform Pharmacotherapeutic Response**

**Online Supplement**

**Subgroup analysis**

Based on the multidimensional scaling (Figure S1A) and edge similarity data (Figure S5B), three subclusters were identified by using hierarchical clustering. Then subgroup-level analyses (e.g., identifying DEGs, dysregulated pathways) were conducted and the clinical data were linked to individualized interactomes stratified by clusters. The differential gene expression analysis was conducted on the patients in each cluster vs. the controls. A ‘consensus’ network was constructed for each cluster by including the edges present in >50% of patients in the cluster. According to the hypergeometric test, the consensus networks are significantly enriched with differential expression genes (DEGs). Pathway analyses were conducted on the proteins involved in the edges present in 90% of patients in the clusters.

**Topological analysis and endophenotype enrichment of sample-specific networks**

Topological analysis of the individualized PAH interactomes shows that while all the networks have similar density, centralization, and heterogeneity, larger networks tend to have a smaller number of connected components, larger size of the largest connected component, and shorter diameters and characteristic path length (**Supplemental Table S5**). The principal clinical features of PAH are established^1^. We collected genes associated with 75 representative endophenotypes that might be relevant to PAH and also have more than 20 associated genes from Phenopedia^2^, DisGeNet^3^, and OpenTargets^4^. Since hypoxia, oxidative stress, angiogenesis, cell proliferation, and immune response are common endophenotypes in cardiovascular diseases, these were added to the endophenotype list. We compiled hypoxia-responsive genes from our in-house microarray gene expression data and a public microarray dataset^5^. Genes associated with oxidative stress, angiogenesis, cell proliferation, apoptosis, immune response were extracted from the Gene Ontology (GO) database (http://geneontology.org/) by using the corresponding GO terms. In total, 75 endophenotypes were considered. We next used the hypergeometric test to determine whether an individualized PAH interactome is significantly enriched with genes associated with these endophenotypes compared to the human interactome as the background.

**Network sensitivity analysis**

Although the number of control samples available for individualized interactome assembly was limited, the statistical framework used to evaluate the significance (p-value) of Δ*r,* explicitly accounts for the number of control samples (n), thereby partially mitigating concerns related to small sample size. To further assess the robustness of interactome assembly, we performed sensitivity analyses by generating synthetic samples using both a gene-wise min–max approach and a constrained subspace method based on convex combinations of the original controls, which produced comparable results. We then reconstructed networks with and without these synthetic samples and quantified their similarity using the overlap coefficient. As shown in Figure S9, networks constructed after adding one, two, or three synthetic samples exhibited substantial topological agreement with the original networks. Given the large edge space, this level of agreement suggests that the overall network structure is reasonably stable.

| **Table S1.** Primary clinical indication and treatment condition for pharmacotherapies actively prescribed to participants in the current study. | | |
| --- | --- | --- |
| **Pharmacotherapy** | **Primary Clinical Indication** | **Treatment Condition** |
| Ambrisentan | PAH | Interventional Drug |
| Macitentan | PAH | Interventional Drug |
| Tadalafil | PAH | Interventional Drug |
| Sildenafil | PAH | Interventional Drug |
| Treprostinil | PAH | Interventional Drug |
| Selexipag | PAH | Interventional Drug |
| Nifedipine | PAH | Interventional Drug |
| Dehydroepiandrosterone | PAH | Investigational Drug |
| Sotatercept | PAH | Investigational Drug* |
| Venlafaxine | Serotonin Psychotropic | Active Comparator |
| Fluoxetine | Serotonin Psychotropic | Active Comparator |
| Quetiapine | Serotonin Psychotropic | Active Comparator |
| Escitalopram | Serotonin Psychotropic | Active Comparator |
| Desvenlafaxine | Serotonin Psychotropic | Active Comparator |
| Mirtazipine | Serotonin Psychotropic | Active Comparator |
| Citalopram | Serotonin Psychotropic | Active Comparator |
| Bupropion | Non-Serotonin Psychotropic | Inactive Comparator |
| Clonazepam | Non-Serotonin Psychotropic | Inactive Comparator |
| Alprazolam | Non-Serotonin Psychotropic | Inactive Comparator |
| Lorazepam | Non-Serotonin Psychotropic | Inactive Comparator |
| Furosemide | Diuretic | Inactive Comparator |
| Torsemide | Diuretic | Inactive Comparator |
| Metolazone | Diuretic | Inactive Comparator |
| *At the time of this study. Active comparator=biologically plausible to have overlap with the PAH module. Inactive comparator=mechanism of action not known to directly impact PAH and no overlap in the PAH module. PAH=pulmonary arterial hypertension. | | |

| **Table S2. A list of all drugs analyzed in the current study and their corresponding protein targets from Drugbank.** | |
| --- | --- |
| **Drug** | **Drugbank targets** |
| Ambrisentan | EDNRA, EDNRB |
| Bosentan | EDNRA, EDNRB |
| Macitentan | EDNRA, EDNRB |
| Tadalafil | PDE5A, PDE11A |
| Sildenafil | PDE5A, PDE6G, PDE6H |
| Epoprostenol | P2RY12, PTGIR, PGTIS |
| Iloprost | PTGIR, PTGER1, PDE4A, PDE4B, PDE4C, PDE4D, PLAT, PTGDR2 |
| Treprostinil | PTGIR, PPARD, P2RY12 |
| Riociguat | GUCY1A2 |
| Selexipag | PTGIR |
| Diltiazem | CACNG1 |
| Nicardipine | CACNA1C, CACNB2, CACNA1D, CACNA2D1, PDE1A, PDE1B, ADRA1A, ADRA1B, CHRM1, CHRM2, CHRM3, CHRM4, CALM1 |
| Nifedipine | CACNA1C, CACNA2D1, CACNB2, CACNA1D, CACNA1S, CALM1, KCNA1, CACNA1H, NR1I2 |
| Amlodipine | CACNA1C, CACNA2D1, CACNB2, CACNA1D, CACNA1S, CACNA1B, CACNA2D3, CA1, SMPD1 |
| Dehydroepiandrosterone | ESR1, ESR2, GABRA1, GABRA3, GABRA4, GABRA5, GABRA6, GABRB1, GABRB2, GABRB3, GABRD, GABRE, GABRG1, GABRG2, GABRQ, GRIN1, GRIN2A, GRIN2B, GRIN2C, GRIN2D, GRIN3A, GRIN3B, AR, HSD17B1, SULT2A1, SULT2B1, PPARA, SIGMAR1, NR1I2, NR1I3 |
| Sotatercept | ACVR2A |
| Venlafaxine | SLC6A4, SLC6A2, SLC6A3, |
| Fluoextine | SLC6A4, KCNH2, HTR2C, CKS1B |
| Quetiapine | HTR2A, DRD2, HTR1A, HTR1B, HTR1E, HTR2C, HTR3A, HTR6, DRD1, DRD5, DRD3, DRD4, HRH1, ADRA1A, ADRA1B, ADRA2A, ADRA2B, ADRA2C, CHRM1, CHRM2, CHRM3, CHRM4 |
| Escitalopram | SLC6A4, SLC6A3, SLC6A2, ADRA1A, CHRM1, HRH1 |
| Desvenlafaxine | SLC6A4, SLC6A2 |
| Mirtazipine | HTR2A, HTR3A, ADRA2A, HTR2C, OPRK1, HRH1, ADRA2C, ADRA1A, ADRA1B, DRD2, DRD3, SLC6A2, SLC6A4, SLC6A3, HTR2B, DRD1, DRD5, ADRB1, ADRB2 |
| Citalopram | SLC6A4, HRH1, ADRA1A, CHRM1, HTR2C |
| Bupropion | SLC6A3, SLC6A2, CHRNA3 |
| Clonazepam | GABRA1, GABRA3, GABRA4, GABRA5, GABRA6, GABRB1, GABRB2, GABRB3, GABRD, GABRE, GABRG1, GABRG2, GABRQ, NR1I2 |
| Alprazolam | GABRA1, GABRA3, GABRA4, GABRA5, GABRA6, GABRB1, GABRB2, GABRB3, GABRD, GABRE, GABRG1, GABRG2, GABRQ |
| Lorazepam | GABRA1, GABRA3, GABRA4, GABRA5, GABRA6, GABRB1, GABRB2, GABRB3, GABRD, GABRE, GABRG1, GABRG2, GABRQ |
| Furosemide | SLC12A1, CA2 |
| Torsemide | SLC12A1 |
| Bumetanide | SLC12A1, SLC12A2, SLC12A4, SLC12A5, CFTR |
| Metolazone | SLC12A3 |

| **Table S3. REVEAL 2.0 scores at 6- and 12-months post-right heart catheterization (RHC) and pharmacologic therapy pre- and post-RHC.** | | | | | | | |
| --- | --- | --- | --- | --- | --- | --- | --- |
| **Patient** | **Age** | **Sex** | **PAH Subtype** | **REVEAL 2.0  6 mos post-RHC** | **REVEAL 2.0  12 mos post-RHC** | **Treatment Before RHC** | **Treatment After RHC** |
| P001 | 51 | F | HIV | 10 | 7 | PDE5i, PA | PDE5i, ERA, PA |
| P002 | 37 | M | IPAH | 5 | 5 | PDE5i | PDE5i, ERA, PA |
| P003 | 74 | F | CHD | 8 | 6 | ERA | ERA |
| P004 | 36 | F | IPAH | 8 | 3 | PDE5i, CCB, ERA | PDE5i, CCB, ERA |
| P005 | 56 | F | PoPH | 7 | 8 | PDE5i, ERA, PA | None* |
| P006 | 56 | F | EiPH | 5 | 5 | None | ERA |
| P007 | 83 | F | IPAH | 11 | 10 | None | PDE5i |
| P008 | 42 | F | IPAH | 10 | 7 | PDE5i, ERA | PDE5i, ERA, PA, DHEA, Sotatercept |
| P009 | 76 | F | IPAH | 9 | 8 | None | PDE5i, ERA, PA |
| P010 | 44 | M | CTD | 12 | 9 | PDE5i, ERA, PA | PDE5i, ERA, PA |
| P011 | 34 | F | HPAH | . | . | None | None |
| P012 | 68 | F | CTD | 12 | 11 | PDE5i | PDEi, ERA, PA |
| P013 | 67 | F | CTD | 12 | 11 | PDE5i, PA | PDE5i, PA |
| P014 | 61 | F | CHD | 9 | 3 | PDE5i | PDE5i |
| P015 | 68 | F | CTD | . | . | None | None |
| P016 | 53 | F | EiPH | 6 | 5 | None | ERA |
| P017 | 30 | F | HPAH | 11 | 6 | PDE5i, ERA, PA | PDE5i, ERA, PA |
| P018 | 68 | F | CTD | 8 | 8 | PDE5i, ERA | PDE5i, ERA, PA |
| P019 | 67 | F | PVOD | 10 | 10 | None | PDE5i |
| P020 | 52 | F | IPAH | 10 | 9 | None | PDE5i, ERA, PA |
| P021 | 50 | F | IPAH | 6 | 4 | PDE5i | PDE5i, ERA |
| P022 | 74 | F | CTD | 14 | 12 | None | PDE5i |
| P023 | 74 | F | CTD | 10 | 7 | None | PA |
| P024 | 46 | F | HPAH | 8 | 7 | PDE5i, ERA | PDE5i, ERA, DHEA |
| P025 | 84 | F | PVOD | 12 | 11 | PDE5i | PDE5i, PA |
| PAH=pulmonary arterial hypertension. REVEAL= Registry to Evaluate Early and Long-Term PAH Disease Management. RHC=right heart catheterization. F=female biologic sex. M=male biologic sex. HIV=human immunodeficiency virus associated PAH. IPAH=idiopathic PAH. CHD=congenital heart disease associated PAH. PoPH=portopulmonary hypertension. EiPH=exercise-induced pulmonary hypertension. CTD=connective tissue disease associated PAH. HPAH=hereditary PAH. PVOD=pulmonary veno-occlusive disease. PDE5i=phosphodiesterase 5 inhibitor. PA=prostacyclin agonist. ERA=endothelin receptor antagonist. CCB=calcium channel blocker. DHEA=dehydroepiandrosterone. *=status post liver transplant. | | | | | | | |

| **Table S4. Topological features of individualized PAH interactomes.** | | | | | | | | | | |
| --- | --- | --- | --- | --- | --- | --- | --- | --- | --- | --- |
|  | **Nodes**  **(n)** | **Edges**  **(n)** | **Connected Components**  **(n)** | **Size of the largest connected component** | **Network diameter** | **Characteristic path length** | **Average number of neighbors** | **Network centralization** | **Network density** | **Network heterogeneity** |
| **P005E** | 7619 | 13914 | 92 | 7413 | 18 | 6.204 | 3.723 | 0.010 | 0.001 | 1.318 |
| **P014** | 7989 | 16492 | 94 | 7768 | 18 | 5.915 | 4.213 | 0.016 | 0.001 | 1.540 |
| **P010B** | 8027 | 16961 | 94 | 7811 | 17 | 5.796 | 4.311 | 0.016 | 0.001 | 1.582 |
| **P001** | 6594 | 10091 | 132 | 6276 | 17 | 6.602 | 3.156 | 0.014 | 0.001 | 1.433 |
| **P004** | 7257 | 14259 | 90 | 7055 | 16 | 6.050 | 4.010 | 0.014 | 0.001 | 1.444 |
| **P007** | 7446 | 13550 | 98 | 7231 | 17 | 6.121 | 3.715 | 0.020 | 0.001 | 1.581 |
| **P008** | 7654 | 14228 | 86 | 7455 | 16 | 6.128 | 3.786 | 0.012 | 0.001 | 1.331 |
| **P010A** | 7347 | 12724 | 91 | 7135 | 18 | 6.315 | 3.532 | 0.018 | 0.001 | 1.376 |
| **P011** | 6704 | 10756 | 119 | 6422 | 16 | 6.484 | 3.299 | 0.011 | 0.001 | 1.329 |
| **P012** | 6642 | 10347 | 105 | 6407 | 17 | 6.590 | 3.189 | 0.014 | 0.001 | 1.391 |
| **P017** | 2753 | 2950 | 112 | 2388 | 23 | 7.562 | 2.257 | 0.036 | 0.001 | 2.302 |
| **P016** | 4027 | 4813 | 124 | 3677 | 27 | 7.139 | 2.494 | 0.026 | 0.001 | 2.213 |
| **P015** | 2313 | 2369 | 122 | 1939 | 28 | 9.091 | 2.183 | 0.038 | 0.001 | 2.123 |
| **P005D** | 3874 | 4354 | 172 | 3415 | 24 | 7.812 | 2.318 | 0.027 | 0.001 | 1.983 |
| **P002** | 3029 | 3285 | 148 | 2615 | 21 | 7.510 | 2.308 | 0.044 | 0.001 | 2.347 |
| **P003** | 3119 | 3448 | 123 | 2773 | 21 | 7.419 | 2.354 | 0.032 | 0.001 | 2.239 |
| **P005A** | 2737 | 2914 | 134 | 2347 | 20 | 7.687 | 2.264 | 0.036 | 0.001 | 2.145 |
| **P005B** | 2905 | 3078 | 150 | 2456 | 21 | 8.230 | 2.262 | 0.032 | 0.001 | 2.040 |
| **P005C** | 2609 | 2763 | 120 | 2269 | 26 | 8.519 | 2.241 | 0.031 | 0.001 | 2.114 |
| **P006** | 2074 | 2097 | 134 | 1611 | 26 | 8.494 | 2.194 | 0.043 | 0.001 | 2.010 |
| **P009** | 2428 | 2628 | 80 | 2165 | 20 | 7.288 | 2.257 | 0.048 | 0.001 | 2.526 |
| **P013** | 3350 | 3939 | 100 | 6680 | 18 | 6.975 | 2.448 | 0.031 | 0.001 | 2.142 |
| **P012B** | 7685 | 14396 | 112 | 7436 | 16 | 5.983 | 3.835 | 0.022 | 0.001 | 1.620 |
| **P018** | 7231 | 12913 | 114 | 6977 | 18 | 6.24 | 3.661 | 0.021 | 0.001 | 1.698 |
| **P019** | 5135 | 6632 | 164 | 4729 | 20 | 7.06 | 2.702 | 0.032 | 0.001 | 1.999 |
| **P020** | 5313 | 6896 | 176 | 4885 | 20 | 7.223 | 2.72 | 0.019 | 0.001 | 1.631 |
| **P021** | 7254 | 12650 | 114 | 6995 | 17 | 6.304 | 3.575 | 0.012 | 0.001 | 1.505 |
| **P022** | 8828 | 20023 | 95 | 8620 | 15 | 5.629 | 4.619 | 0.018 | 0.001 | 1.407 |
| **P008B** | 4820 | 5972 | 164 | 4397 | 20 | 7.382 | 2.598 | 0.024 | 0.001 | 1.787 |
| **P023** | 9569 | 24877 | 78 | 9404 | 15 | 5.465 | 5.272 | 0.012 | 0.001 | 1.234 |
| **P024** | 7855 | 15180 | 91 | 7667 | 15 | 5.867 | 3.934 | 0.015 | 0.001 | 1.567 |
| **P025** | 5215 | 6711 | 150 | 4823 | 18 | 7.061 | 2.682 | 0.016 | 0.001 | 1.771 |
|  | | | | | | | | | | |

| **Table S5. Characteristics of PAH patient subclusters and corresponding consensus interactomes.** | | | |
| --- | --- | --- | --- |
|  | Cluster 1 | Cluster 2 | Cluster 3 |
| DEGs | 21 | 1151 | 1072 |
| Consensus Interactome | 3682 nodes  3681 edges | 1492 nodes  1421 edges | 2909 nodes  2829 edges |
| Enrichment of DEGs in the consensus interactome | 10  2.5-fold  p < 0.003 | 195  2.0-fold  p < 0.001 | 286  1.6-fold  p < 0.001 |
| Age, years | 48 (34 – 83) | 56 (30 – 76) | 67 (46 – 84) |
| Female sex, n (%) | 8 (80) | 11 (92) | 10 (100) |
| BMI, kg/m^2^ | 30 (22 – 44) | 29 (16 – 43) | 28 (22 – 45) |
| PAH subtype |  | | |
| Idiopathic PAH, n (%) | 3 (30) | 2 (17) | 3 (30) |
| Connective tissue disease PAH, n (%) | 3 (30) | 2 (17) | 4 (40) |
| Other PAH, n (%) | 4 (40) | 8 (67) | 3 (70) |
| mPAP, mm Hg | 38 (23 – 57) | 33 (16 – 62) | 47 (26 – 69) |
| PVR, Wood Units | 4.0 (1.1 – 9.3) | 3.3 (1.0 – 19.6) | 4.8 (1.1 – 18.7) |
| REVEAL 2.0 risk | 5 (3 – 11) | 9 (4 – 14) | 8 (5 – 12) |
| All values expressed as median (range) or n (%) unless otherwise indicated. DEG=differentially expressed gene. SD=standard deviation. BMI=body mass index. PAH=pulmonary arterial hypertension. mPAP=mean pulmonary artery pressure. PVR=pulmonary vascular resistance. REVEAL= Registry to EValuate Early And Long-term pulmonary arterial hypertension disease management. | | | |

| **Table S6. Rank order of PAH therapeutic targets in the individualized interactomes.** | | |
| --- | --- | --- |
| **Total number of PAH targets in the corresponding individualized Interactome** | **Participant ID Code** | **Molecular Targets** |
| **21** | **P023** | ADRA1B, CACNA1H, CACNA2D1, CACNB2, CALM1, GUCY1A2, EDNRA, EDNRB, PDE4A, PDE4B, PDE4D, PLAT, PPARD, PTGIR, ACVR2A, AR, GABRE, GRIN3B, NR1I3, PPARA, SIGMAR1 |
| **19** | **P007** | ADRA1B, CACNA1H, CACNA2D1, CALM1, PDE1A, PDE5A, EDNRA, PDE4B, PDE4D, PLAT, PPARD, PTGIR, ACVR2A, AR, GABRE, GRIN3B, HSD17B1, PPARA, SIGMAR1 |
| **18** | **P014** | CACNA1H, CACNA2D1, CACNB2, CALM1, EDNRA, EDNRB, PDE4D, PLAT, PPARD, PTGIR, ACVR2A, AR, GABRB3, GABRE, GRIN2D, GRIN3B, PPARA, SIGMAR1 |
| **18** | **P010A** | ADRA1B, CACNA1H, CACNB2, CALM1, PDE5A, EDNRA, EDNRB, PLAT, PPARD, PTGIR, ACVR2A, AR, GABRE, GRIN2D, GRIN3B, HSD17B1, PPARA, SIGMAR1 |
| **18** | **P021** | CACNA1C, CACNA2D1, CACNB2, CALM1, EDNRA, EDNRB, PDE4A, PDE4B, PDE4D, PLAT, PPARD, PTGIR, ACVR2A, AR, GABRE, HSD17B1, PPARA, SIGMAR1 |
| **17** | **P010B** | ADRA1B, CACNA1H, CACNA2D1, CACNB2, CALM1, EDNRA, PDE4D, PLAT, PPARD, PTGIR, ACVR2A, AR, GABRE, GRIN2D, GRIN3B, HSD17B1, SIGMAR1 |
| **17** | **P012B** | CACNB2, CALM1, EDNRB, PDE4A, PDE4B, PDE4D, PLAT, PPARD, PTGIR, ACVR2A, AR, GABRB3, GRIN2D, GRIN3B, NR1I3, PPARA, SIGMAR1 |
| **17** | **P022** | ADRA1B, CACNA1H, CACNA2D1, CACNB2, CALM1, EDNRA, EDNRB, PDE4B, PDE4D, PPARD, PTGIR, ACVR2A, AR, GRIN2D, HSD17B1, PPARA, SIGMAR1 |
| **17** | **P024** | CACNA1C, CACNB2, CALM1, PDE5A, EDNRA, PDE4A, PDE4B, PDE4D, PPARD, PTGIR, ACVR2A, AR, GABRB3, GABRE, NR1I3, PPARA, SIGMAR1 |
| **16** | **P005E** | ADRA1B, CACNA2D1, CACNB2, CALM1, PDE1A, EDNRB, PDE4B, PDE4D, PLAT, PPARD, PTGIR, ACVR2A, AR, GABRE, GRIN3B, PPARA |
| **16** | **P011** | CACNA1H, CACNA2D1, CALM1, PDE5A, EDNRA, PDE4D, PLAT, PPARD, PTGIR, ACVR2A, AR, GABRE, GRIN2D, GRIN3B, PPARA, SIGMAR1 |
| **16** | **P020** | CACNA1H, CACNA2D1, CACNB2, CALM1, GUCY1A2, EDNRA, EDNRB, PDE4B, PLAT, PPARD, PTGIR, ACVR2A, GABRE, HSD17B1, PPARA, SIGMAR1 |
| **16** | **P025** | ADRA1B, CACNA1H, CACNA2D1, CACNB2, CALM1, EDNRA, PDE4D, PLAT, PPARD, PTGIR, ACVR2A, AR, GABRE, HSD17B1, PPARA, SIGMAR1 |
| **15** | **P004** | ADRA1B, CACNA1H, CACNB2, CALM1, EDNRA, PDE4B, PDE4D, PLAT, PPARD, PTGIR, AR, GABRE, GRIN2D, PPARA, SIGMAR1 |
| **15** | **P008** | CACNA2D1, CACNB2, CALM1, PDE5A, PDE4D, PPARD, PTGIR, ACVR2A, AR, GABRE, GRIN3B, HSD17B1, NR1I3, PPARA, SIGMAR1 |
| **15** | **P012** | ADRA1B, CACNA2D1, CACNB2, CALM1, EDNRA, EDNRB, PDE4A, PDE4D, PLAT, PPARD, PTGIR, AR, GABRE, PPARA, SIGMAR1 |
| **14** | **P018** | ADRA1B, CACNA2D1, CALM1, EDNRA, PDE4B, PDE4D, PPARD, PTGIR, ACVR2A, AR, GABRE, HSD17B1, PPARA, SIGMAR1 |
| **14** | **P019** | ADRA1B, CACNA1H, CACNA2D1, EDNRA, PDE4D, PLAT, PPARD, ACVR2A, AR, GABRE, GRIN3B, HSD17B1, PPARA, SIGMAR1 |
| **13** | **P001** | ADRA1B, CACNA2D1, CACNB2, CALM1, PDE1A, EDNRA, PLAT, PPARD, PTGIR, AR, GABRE, PPARA, SIGMAR1 |
| **13** | **P003** | CACNA1H, CACNA2D1, CALM1, GUCY1A2, EDNRA, PLAT, PPARD, PTGIR, ACVR2A, HSD17B1, NR1I3, PPARA, SIGMAR1 |
| **12** | **P017** | ADRA1B, CACNA1H, CALM1, GUCY1A2, PLAT, PPARD, PTGIR, ACVR2A, AR, GRIN3B, HSD17B1, SIGMAR1 |
| **12** | **P005D** | CACNA1H, CALM1, EDNRA, PLAT, PPARD, PTGIR, ACVR2A, AR, GABRB3, GRIN2D, HSD17B1, SIGMAR1 |
| **12** | **P005B** | CACNA2D1, CALM1, EDNRB, PLAT, PTGIR, ACVR2A, GABRB3, GABRE, GRIN3B, HSD17B1, PPARA, SIGMAR1 |
| **12** | **P008B** | CACNA1H, CACNB2, CALM1, PDE1A, EDNRA, PLAT, PPARD, ACVR2A, AR, GABRE, HSD17B1, SIGMAR1 |
| **11** | **P005A** | CACNA1C, CACNA1H, CALM1, EDNRA, EDNRB, PLAT, ACVR2A, GABRB3, GRIN3B, HSD17B1, SIGMAR1 |
| **11** | **P013** | ADRA1B, CACNA1H, CACNA2D1, CALM1, PDE1A, EDNRA, PDE4D, PLAT, AR, GABRB3, SIGMAR1 |
| **9** | **P002** | CACNA1C, CACNA1H, CALM1, EDNRB, PLAT, PTGIR, AR, GABRB3, SIGMAR1 |
| **8** | **P006** | CALM1, EDNRB, PLAT, PPARD, ACVR2A, GABRB3, HSD17B1, SIGMAR1 |
| **7** | **P016** | CACNA2D1, CALM1, PLAT, AR, GABRB3, PPARA, SIGMAR1 |
| **6** | **P015** | CACNA1H, CALM1, EDNRB, PLAT, ACVR2A, SIGMAR1 |
| **6** | **P009** | CACNA2D1, CALM1, PLAT, PPARD, AR, GABRB3 |
| **5** | **P005C** | CACNA1H, CACNA2D1, PDE1A, GABRB3, SIGMAR1 |

| **Table S7. Rank order of PAH therapeutic targets in individualized interactomes stratified by drug class.** | | | | | | | | |
| --- | --- | --- | --- | --- | --- | --- | --- | --- |
| **Total number of PAH targets in the corresponding individualized interactomes** | **Patient**  **Participant ID code** | **Molecular Targets** | | | | | | |
|  |  | **Calcium Channel Blockers** | **PDE5i** | **sGC** | **Endothelin Receptor Antagonist** | **Prostacyclin** | **Sotatercept** | **DHEA** |
| 21 | P023 | ADRA1B, CACNA1H, CACNA2D1, CACNB2, CALM1 |  | GUCY1A2 | EDNRA, EDNRB | PDE4A, PDE4B, PDE4D, PLAT, PPARD, PTGIR | ACVR2A | AR, GABRE, GRIN3B, NR1I3, PPARA, SIGMAR1 |
| 19 | P007 | ADRA1B, CACNA1H, CACNA2D1, CALM1, PDE1A | PDE5A |  | EDNRA | PDE4B, PDE4D, PLAT, PPARD, PTGIR | ACVR2A | AR, GABRE, GRIN3B, HSD17B1, PPARA, SIGMAR1 |
| 18 | P014 | CACNA1H, CACNA2D1, CACNB2, CALM1 |  |  | EDNRA, EDNRB | PDE4D, PLAT, PPARD, PTGIR | ACVR2A | AR, GABRB3, GABRE, GRIN2D, GRIN3B, PPARA, SIGMAR1 |
| 18 | P010A | ADRA1B, CACNA1H, CACNB2, CALM1 | PDE5A |  | EDNRA, EDNRB | PLAT, PPARD, PTGIR | ACVR2A | AR, GABRE, GRIN2D, GRIN3B, HSD17B1, PPARA, SIGMAR1 |
| 18 | P021 | CACNA1C, CACNA2D1, CACNB2, CALM1 |  |  | EDNRA, EDNRB | PDE4A, PDE4B, PDE4D, PLAT, PPARD, PTGIR | ACVR2A | AR, GABRE, HSD17B1, PPARA, SIGMAR1 |
| 17 | P010B | ADRA1B, CACNA1H, CACNA2D1, CACNB2, CALM1 |  |  | EDNRA | PDE4D, PLAT, PPARD, PTGIR | ACVR2A | AR, GABRE, GRIN2D, GRIN3B, HSD17B1, SIGMAR1 |
| 17 | P012B | CACNB2, CALM1 |  |  | EDNRB | PDE4A, PDE4B, PDE4D, PLAT, PPARD, PTGIR | ACVR2A | AR, GABRB3, GRIN2D, GRIN3B, NR1I3, PPARA, SIGMAR1 |
| 17 | P022 | ADRA1B, CACNA1H, CACNA2D1, CACNB2, CALM1 |  |  | EDNRA, EDNRB | PDE4B, PDE4D, PPARD, PTGIR | ACVR2A | AR, GRIN2D, HSD17B1, PPARA, SIGMAR1 |
| 17 | P024 | CACNA1C, CACNB2, CALM1 | PDE5A |  | EDNRA | PDE4A, PDE4B, PDE4D, PPARD, PTGIR | ACVR2A | AR, GABRB3, GABRE, NR1I3, PPARA, SIGMAR1 |
| 16 | P005E | ADRA1B, CACNA2D1, CACNB2, CALM1, PDE1A |  |  | EDNRB | PDE4B, PDE4D, PLAT, PPARD, PTGIR | ACVR2A | AR, GABRE, GRIN3B, PPARA |
| 16 | P011 | CACNA1H, CACNA2D1, CALM1 | PDE5A |  | EDNRA | PDE4D, PLAT, PPARD, PTGIR | ACVR2A | AR, GABRE, GRIN2D, GRIN3B, PPARA, SIGMAR1 |
| 16 | P020 | CACNA1H, CACNA2D1, CACNB2, CALM1 |  | GUCY1A2 | EDNRA, EDNRB | PDE4B, PLAT, PPARD, PTGIR | ACVR2A | GABRE, HSD17B1, PPARA, SIGMAR1 |
| 16 | P025 | ADRA1B, CACNA1H, CACNA2D1, CACNB2, CALM1 |  |  | EDNRA | PDE4D, PLAT, PPARD, PTGIR | ACVR2A | AR, GABRE, HSD17B1, PPARA, SIGMAR1 |
| 15 | P004 | ADRA1B, CACNA1H, CACNB2, CALM1 |  |  | EDNRA | PDE4B, PDE4D, PLAT, PPARD, PTGIR |  | AR, GABRE, GRIN2D, PPARA, SIGMAR1 |
| 15 | P008 | CACNA2D1, CACNB2, CALM1 | PDE5A |  |  | PDE4D, PPARD, PTGIR | ACVR2A | AR, GABRE, GRIN3B, HSD17B1, NR1I3, PPARA, SIGMAR1 |
| 15 | P012 | ADRA1B, CACNA2D1, CACNB2, CALM1 |  |  | EDNRA, EDNRB | PDE4A, PDE4D, PLAT, PPARD, PTGIR |  | AR, GABRE, PPARA, SIGMAR1 |
| 14 | P018 | ADRA1B, CACNA2D1, CALM1 |  |  | EDNRA | PDE4B, PDE4D, PPARD, PTGIR | ACVR2A | AR, GABRE, HSD17B1, PPARA, SIGMAR1 |
| 14 | P019 | ADRA1B, CACNA1H, CACNA2D1 |  |  | EDNRA | PDE4D, PLAT, PPARD | ACVR2A | AR, GABRE, GRIN3B, HSD17B1, PPARA, SIGMAR1 |
| 13 | P001 | ADRA1B, CACNA2D1, CACNB2, CALM1, PDE1A |  |  | EDNRA | PLAT, PPARD, PTGIR |  | AR, GABRE, PPARA, SIGMAR1 |
| 13 | P003 | CACNA1H, CACNA2D1, CALM1 |  | GUCY1A2 | EDNRA | PLAT, PPARD, PTGIR | ACVR2A | HSD17B1, NR1I3, PPARA, SIGMAR1 |
| 12 | P017 | ADRA1B, CACNA1H, CALM1 |  | GUCY1A2 |  | PLAT, PPARD, PTGIR | ACVR2A | AR, GRIN3B, HSD17B1, SIGMAR1 |
| 12 | P005D | CACNA1H, CALM1 |  |  | EDNRA | PLAT, PPARD, PTGIR | ACVR2A | AR, GABRB3, GRIN2D, HSD17B1, SIGMAR1 |
| 12 | P005B | CACNA2D1, CALM1 |  |  | EDNRB | PLAT, PTGIR | ACVR2A | GABRB3, GABRE, GRIN3B, HSD17B1, PPARA, SIGMAR1 |
| 12 | P008B | CACNA1H, CACNB2, CALM1, PDE1A |  |  | EDNRA | PLAT, PPARD | ACVR2A | AR, GABRE, HSD17B1, SIGMAR1 |
| 11 | P005A | CACNA1C, CACNA1H, CALM1 |  |  | EDNRA, EDNRB | PLAT | ACVR2A | GABRB3, GRIN3B, HSD17B1, SIGMAR1 |
| 11 | P013 | ADRA1B, CACNA1H, CACNA2D1, CALM1, PDE1A |  |  | EDNRA | PDE4D, PLAT |  | AR, GABRB3, SIGMAR1 |
| 9 | P002 | CACNA1C, CACNA1H, CALM1 |  |  | EDNRB | PLAT, PTGIR |  | AR, GABRB3, SIGMAR1 |
| 8 | P006 | CALM1 |  |  | EDNRB | PLAT, PPARD | ACVR2A | GABRB3, HSD17B1, SIGMAR1 |
| 7 | P016 | CACNA2D1, CALM1 |  |  |  | PLAT |  | AR, GABRB3, PPARA, SIGMAR1 |
| 6 | P015 | CACNA1H, CALM1 |  |  | EDNRB | PLAT | ACVR2A | SIGMAR1 |
| 6 | P009 | CACNA2D1, CALM1 |  |  |  | PLAT, PPARD |  | AR, GABRB3 |
| 5 | P005C | CACNA1H, CACNA2D1, PDE1A |  |  |  |  |  | GABRB3, SIGMAR1 |

| **Table S8. Rank order of therapeutic targets for diuretic comparators in the individualized interactomes.** | | |
| --- | --- | --- |
| **Total number of diuretic comparator targets in corresponding individualized interactomes** | **Participant ID code** | **Molecular targets** |
| 3 | P005E | CA2, SLC12A2, SLC12A4 |
| 3 | P014 | CA2, SLC12A2, SLC12A4 |
| 3 | P001 | CA2, SLC12A2, SLC12A4 |
| 3 | P004 | CA2, SLC12A2, SLC12A4 |
| 3 | P007 | CA2, SLC12A2, SLC12A4 |
| 3 | P008 | CA2, SLC12A2, SLC12A4 |
| 3 | P010A | CA2, SLC12A2, SLC12A4 |
| 3 | P011 | CA2, SLC12A2, SLC12A4 |
| 3 | P012 | CA2, SLC12A2, SLC12A4 |
| 3 | P017 | CA2, SLC12A2, SLC12A4 |
| 3 | P016 | CA2, SLC12A2, SLC12A4 |
| 3 | P015 | CA2, SLC12A2, SLC12A4 |
| 3 | P002 | CA2, SLC12A4, SLC12A2 |
| 3 | P005A | CA2, SLC12A4, SLC12A2 |
| 3 | P012B | CA2, SLC12A4, SLC12A2 |
| 3 | P020 | CA2, SLC12A4, SLC12A2 |
| 3 | P021 | CA2, SLC12A4, SLC12A2 |
| 3 | P008B | CA2, SLC12A4, SLC12A2 |
| 3 | P023 | CA2, SLC12A2, SLC12A4 |
| 3 | P024 | CA2, SLC12A4, SLC12A2 |
| 2 | P010B | SLC12A2, SLC12A4 |
| 2 | P005D | SLC12A2, SLC12A4 |
| 2 | P003 | SLC12A2, SLC12A4 |
| 2 | P005B | SLC12A2, SLC12A4 |
| 2 | P005C | SLC12A2, SLC12A4 |
| 2 | P006 | SLC12A2, SLC12A4 |
| 2 | P009 | SLC12A4, SLC12A2 |
| 2 | P013 | SLC12A4, SLC12A2 |
| 2 | P018 | CA2, SLC12A4 |
| 2 | P019 | CA2, SLC12A4, |
| 2 | P022 | SLC12A4, SLC12A2 |
| 2 | P025 | CA2, SLC12A4 |
|  | | |

| **Table S9. Rank order of therapeutic targets for serotonergic psychotropic comparators in the individualized interactomes.** | | |
| --- | --- | --- |
| **Total number of** serotonergic **psychotropic comparators** **targets in corresponding individualized interactomes** | **Participant ID code** | **Serotonergic**  **Molecular targets** |
| 7 | P019 | CKS1B, SLC6A4, HTR1B, ADRA1B, ADRA2A, ADRB1, ADRB2 |
| 7 | P023 | CKS1B, HRH1, ADRA1B, HTR1B, ADRA2A, ADRB2, HTR2B |
| 7 | P025 | CKS1B, HRH1, HTR1B, ADRA1B, ADRA2A, ADRB2, ADRB1 |
| 6 | P010B | CKS1B, SLC6A4, ADRA1B, HTR1B, HRH1, ADRB2 |
| 6 | P007 | CKS1B, SLC6A4, ADRA1B, HTR1B, HRH1, ADRB2 |
| 6 | P002 | CKS1B, SLC6A4, ADRA1B, ADRA2A, HTR1B, ADRB2, |
| 6 | P018 | CKS1B, HTR1B, HRH1, ADRA1B, ADRB1, ADRB2 |
| 6 | P024 | CKS1B, SLC6A4, HTR1B, ADRA2A, HTR2B, ADRB2 |
| 5 | P005E | CKS1B, ADRA1B, HTR1B, HRH1, ADRB2 |
| 5 | P014 | CKS1B, ADRA1B, HTR1B, HRH1, ADRB2 |
| 5 | P001 | CKS1B, ADRA1B, HTR1B, HRH1, ADRB2 |
| 5 | P004 | CKS1B, ADRA1B, HTR1B, HRH1, ADRB2 |
| 5 | P008 | CKS1B, HTR1B, ADRA1B, HRH1, ADRB2 |
| 5 | P010A | CKS1B, ADRA1B, HTR1B, HRH1, ADRB2 |
| 5 | P011 | CKS1B, ADRA1B, HTR1B, HRH1, ADRB2 |
| 5 | P012 | CKS1B, ADRA1B, HTR1B, HRH1, ADRB2 |
| 5 | P017 | CKS1B, ADRA1B, HTR1B, HRH1, ADRB2 |
| 5 | P016 | CKS1B, ADRA1B, HTR1B, HRH1, ADRB2 |
| 5 | P015 | CKS1B, ADRA1B, HTR1B, HRH1, ADRB2 |
| 5 | P005D | CKS1B, HTR1B, ADRA1B, ADRA2A, ADRB2 |
| 5 | P003 | CKS1B, SLC6A4, ADRA2A, HTR1B, ADRB2 |
| 5 | P005A | CKS1B, ADRA1B, HTR1B, HRH1, ADRB2 |
| 5 | P005C | CKS1B, HTR1B, ADRA1B, ADRA2A, ADRB2 |
| 5 | P013 | CKS1B, HTR1B, ADRA1B, ADRA2A, ADRB2 |
| 5 | P020 | SLC6A4, HTR1B, ADRA2A, HTR2B, ADRB2 |
| 5 | P021 | CKS1B, SLC6A4, HTR1B, HTR2B, ADRB2 |
| 5 | P022 | CKS1B, ADRA1B, HTR1B, HTR2B, ADRB2 |
| 4 | P005B | CKS1B, HTR1B, HRH1, ADRB2 |
| 4 | P009 | CKS1B, HTR1B, ADRA2A, ADRB2 |
| 4 | P012B | CKS1B, HTR1B, ADRB2, HTR2B |
| 3 | P006 | CKS1B, HTR1B, ADRB2 |
| 3 | P008B | CKS1B, ADRB2, HTR2B |

| **Table S10. Rank order of therapeutic targets for non-serotonergic psychotropic comparators in the individualized interactomes.** | | |
| --- | --- | --- |
| **Total number of** **non-serotonergic psychotropic comparators** **targets in corresponding individualized interactomes** | **Participant ID code** | **Non-Serotonergic**  **Molecular targets** |
| 2 | P024 | GABRE, GABRB3 |
| 1 | P005E | GABRE |
| 1 | P014 | GABRE |
| 1 | P010B | GABRE |
| 1 | P001 | GABRE |
| 1 | P004 | GABRE |
| 1 | P007 | GABRE |
| 1 | P008 | GABRE |
| 1 | P010A | GABRE |
| 1 | P011 | GABRE |
| 1 | P012 | GABRE |
| 1 | P017 | GABRE |
| 1 | P016 | GABRE |
| 1 | P015 | GABRE |
| 1 | P002 | GABRE |
| 1 | P003 | GABRE |
| 1 | P005A | GABRE |
| 1 | P005B | GABRE |
| 1 | P005C | GABRE |
| 1 | P006 | GABRE |
| 1 | P009 | GABRE |
| 1 | P013 | GABRE |
| 1 | P012B | GABRE |
| 1 | P018 | GABRE |
| 1 | P019 | GABRE |
| 1 | P020 | GABRE |
| 1 | P021 | GABRE |
| 1 | P008B | GABRE |
| 1 | P025 | GABRE |
| 0 | P005D |  |
| 0 | P022 |  |
| 0 | P023 |  |
|  | | |

**Figure S1. Pulmonary endothelial transcriptomic profile for PAH samples. A**. The multidimensional scaling plot of the pulmonary artery endothelial cell (PAEC) RNA-Seq dataset. CHD=congenital heart disease associated PAH; CTD=connective tissue disease associated PAH; EiPH=exercise induced pulmonary hypertension; HIV=human immunodeficiency virus associated PAH; HPAH=heritable PAH; IPAH=idiopathic PAH; PoPH=portopulmonary hypertension; PVOD=pulmonary veno-occlusive disease; CTRL=control. **B.** The distribution of the coefficient of variation (CV) in the expression of genes among PAH samples.

**Figure S2.** **Correlation among PAH participant and non-diseased control samples.** The transcript quantity for mRNA isolated from pulmonary artery endothelial cells and measured by RNA-seq were compiled for each control and PAH patient. Pearson’s correlation coefficient between individual control and PAH patients is represented in this table in heatmap form. CHD=congenital heart disease associated PAH; CTD=connective tissue disease associated PAH; EiPH=exercise induced pulmonary hypertension; HIV=human immunodeficiency virus associated PAH; HPAH=heritable PAH; IPAH=idiopathic PAH; PoPH=portopulmonary hypertension; PVOD=pulmonary veno-occlusive disease; CTRL=control.

| **Specimen ID** | **Age (years)** | **Sex** | **PAH Subtype** | **PVR (WU)** |
| --- | --- | --- | --- | --- |
| P005E | 58 | Female | Portopulmonary Hypertension | 4.0 |
| P014 | 61 | Female | Congenital heart disease | 5.3 |
| P010B | 45 | Male | Connective tissue disease | 8.5 |
| P001 | 51 | Female | HIV | 9.3 |
| P004 | 36 | Female | Idiopathic | 2.2 |
| P007 | 83 | Female | Idiopathic | 6.7 |

**Figure S3.** **A sample of individualized PAH interactomes form this study**. Select clinical data is presented in the table above. PAH=pulmonary arterial hypertension; PVR=pulmonary vascular resistance; WU=Wood units; HIV=human immunodeficiency virus.

**Figure S4:** **The mRNA expression profile of PAH therapeutic targets for each individualized interactome.** Heatmap showing the mRNA expression of PAH therapeutic targets in each individualized interactome. The expression of the targets was scaled to: mean 0 and variance 1 cross all the patients.

**A.**

**B.**

**Figure S5. Node (A) and edge (B) overlap similarity between individualized PAH interactomes.**

**A.**

**B.**

**C.**

**Figure S6.** **Pathway enrichment analysis derived from individualized PAH interactomes stratified by patient cluster.** Multidimensional scaling and edge similarity data were used to identify three subclusters using hierarchical clustering. Then subgroup-level analyses were conducted and the clinical data were linked to individualized networks by cluster. Enrichment of differential expression genes (DEGs) were calculated and pathway analyses were conducted on the proteins involved in the edges present in 90% of patients in the clusters (see **Table S3).** The enrichment profile for **(A)** cluster 1, **(B)** cluster 2, and **(C)** cluster 3 are presented.

**Figure S7. Heatmap of endophenotype enrichment in PAH patient samples.** The rows correspond to significant endophenotypes, and the columns denote the cell biopsies from individual participants. The heatmap is depicted as -log_10_*P*, where *P* is the P-value from the endophenotype enrichment analysis after Bonferroni correction.

β 1.9 (95% CI -1.3 to 5.3)

β 2.7 (95% CI 0.3 to 5.0)

**Figure S8**. **Pharmacotherapeutic comparators, target-interactome alignment, and PAH endpoints.** Association was determined using linear regression (β = coefficient) alignment between molecular targets of serotonin-psychotropic comparators and individualized PAH interactomes to United States Registry to Evaluate Early and Long-Term PAH Disease Management (REVEAL) 2.0 risk scores at six (**A**) and twelve months (**B**) after right heart catheterization.

**Figure S9. Stability of network with the addition of synthetic controls.**

**Figure S10.** **The relationship between the number of unique targets per PAH drug class versus the target-interactome alignment**. CCB=calcium channel blocker; PDE5i=phosphodiesterase-type 5 inhibitor; sGC=soluble guanyl cyclase stimulator; ERA=endothelin receptor antagonist.

**References**

1.	Dweik RA, Rounds S, Erzurum SC, et al. An Official American Thoracic Society Statement: Pulmonary Hypertension Phenotypes. *American Journal of Respiratory and Critical Care Medicine* 2014; 189: 345-355. DOI: 10.1164/rccm.201311-1954ST.

2.	Yu W, Clyne M, Khoury MJ, et al. Phenopedia and Genopedia: disease-centered and gene-centered views of the evolving knowledge of human genetic associations. *Bioinformatics* 2010; 26: 145-146. 20091027. DOI: 10.1093/bioinformatics/btp618.

3.	Piñero J, Bravo À, Queralt-Rosinach N, et al. DisGeNET: a comprehensive platform integrating information on human disease-associated genes and variants. *Nucleic Acids Res* 2017; 45: D833-d839. 20161019. DOI: 10.1093/nar/gkw943.

4.	Koscielny G, An P, Carvalho-Silva D, et al. Open Targets: a platform for therapeutic target identification and validation. *Nucleic Acids Res* 2017; 45: D985-d994. 20161129. DOI: 10.1093/nar/gkw1055.

5.	Wang RS, Oldham WM and Loscalzo J. Network-based association of hypoxia-responsive genes with cardiovascular diseases. *New J Phys* 2014; 16: 105014. DOI: 10.1088/1367-2630/16/10/105014.
